# Fucoxanthin Is a Potential Therapeutic Agent for the Treatment of Breast Cancer

**DOI:** 10.3390/md20060370

**Published:** 2022-05-30

**Authors:** Tsz-Ying Lau, Hiu-Yee Kwan

**Affiliations:** Centre for Cancer & Inflammation Research, School of Chinese Medicine, Hong Kong Baptist University, Hong Kong, China; 21482306@life.hkbu.edu.hk

**Keywords:** fucoxanthin, fucoxanthinol, breast cancer, anti-cancer, drug resistance, marine drug, cancer prevention

## Abstract

Breast cancer (BC) is one of the most common cancers diagnosed and the leading cause of cancer-related death in women. Although there are first-line treatments for BC, drug resistances and adverse events have been reported. Given the incidence of BC keeps increasing, seeking novel therapeutics is urgently needed. Fucoxanthin (Fx) is a dietary carotenoid commonly found in seaweeds and diatoms. Both in vitro and in vivo studies show that Fx and its deacetylated metabolite fucoxanthinol (Fxol) inhibit and prevent BC growth. The NF-κB signaling pathway is considered the major pathway contributing to the anti-proliferation, anti-angiogenesis and pro-apoptotic effects of Fx and Fxol. Other signaling molecules such as MAPK, MMP2/9, CYP and ROS are also involved in the anti-cancer effects by regulating the tumor microenvironment, cancer metastasis, carcinogen metabolism and oxidation. Besides, Fx also possesses anti-obesity effects by regulating UCP1 levels and lipid metabolism, which may help to reduce BC risk. More importantly, mounting evidence demonstrates that Fx overcomes drug resistance. This review aims to give an updated summary of the anti-cancer effects of Fx and summarize the underlying mechanisms of action, which will provide novel strategies for the development of Fx as an anti-cancer therapeutic agent.

## 1. Introduction

### 1.1. Breast Cancer Prevalence

Breast cancer (BC) is one of the most common cancers diagnosed and the leading cause of cancer-related death in women [1,2,3]. According to the GLOBOCAN 2020 database, an increasing trend is observed in the global BC cases, and it is estimated that the diagnosed BC cases and mortality rates will be increased by at least 40% in 2040 [4,5]. Therefore, novel and effective therapeutic agents and strategies are emergently needed.

Ductal hyperproliferation is the initiative step of breast tumors’ development. The tumors may develop into benign or malignant tumors after being repeatedly stimulated by carcinogenic factors [6]. There are different molecular/intrinsic subtypes of BCs which could be classified according to histologic information, Nottingham grade, hormone receptor status and HER2 status [7]. Further heterogeneity among BCs was revealed by the RNA analysis, and a total of five major subgroups are identified according to the RNA expression signatures; they are luminal A, luminal B, Her2-enriched, claudin-low and basal-like [8]. ERα-positive BC is mostly related to luminal A and B, while Her2-positive and triple negative breast cancers (TNBCs) are mostly related to Her2-enriched and basal-like, respectively. In general, the aggressiveness of luminal A cancers is lower than luminal B cancers since it has lower expression of Ki67 that is a proliferation marker, and the expression of Her2 in addition to ERα [9]. Not all TNBCs are basal-like subtypes; TNBCs can be further divided into six subtypes with distinct gene expressions and ontologies through cluster analysis, which include basal-like-1 (BL1) and BL2, an immunomodulatory, a mesenchymal, a mesenchymal stem-like and a luminal androgen receptor subtype [10]. The various TNBC subtypes are linked to various outcomes [11].

### 1.2. Genetic Mutations & Clinical Outcomes

Gene expression profiling has confirmed the relationship between BRCA1/2, HER-2, EGFR, Ras and c-Myc genes and the BC initiation and development [6,12]. Inheritance of deleterious mutations in BRCA1/2 genes will significantly increase the risk of having BC; around 25% of hereditary BC and 10% of all the BC are rooted from BRCA1/2 mutations [13,14]. BRCA1-associated BCs mainly shows a basal-like molecular profile [15,16,17], and it appears to occur more frequently in younger patients, and African American women usually have a worse prognosis [18,19], while BRCA2-associated BCs are mainly the luminal phenotype ductal carcinomas with high aggressiveness [20]. The deficiency in BRCA1 will result in cell cycle checkpoint dysregulation, aberrant centrosome duplication, genetic instability and apoptosis [21,22].

HER2 being the oncogene of BC, its overexpression is reported in about 20% of primary BC, which is proved to increase the cancer stem cells’ population via PTEN/Akt/mTORC1 signaling and is associated with poor clinical outcomes [23,24].

EGFR is a member of the tyrosine kinase family, and its overexpression will activate PI3K, Ras-Raf-MAPK (mitogen-activated protein kinases) and JNK signaling pathways, contributing to cancer proliferation, metastasis and the escape from programmed cell death [25,26]. Over 30% of the patients with overexpressed EGFR are diagnosed as inflammatory BC, which has higher aggressiveness and worse prognosis than patients without EGFR overexpression [27,28]. More than half of the TNBC cases are characterized by ER-/PR- with HER2 amplification and EGFR overexpression [29].

Besides, the overexpression of c-Myc in BC promotes protooncogene amplification and affects transcriptional regulation, mRNA and protein stabilities [30], which will promote tumor growth. BC patients with overexpressed c-Myc are usually diagnosed with high-grade invasive carcinomas, while patients without c-Myc amplification are usually diagnosed with benign tissues [31,32].

The overexpression of Ras genes (H-ras, K-ras and N-ras) is also observed in BC, in which mutations occur at the coding domain for the guanosine triphosphate (GTP)-binding protein. Overexpression of H-Ras is common in primary and advanced BC, and it is responsible for BC progression [33,34,35]. The elevation of K-ras found in BC patients is usually associated with more aggressive TNBC while the elevation of N-ras is related to poorer clinical outcome [36].

### 1.3. Clinical Challenges

The combination of Docetaxel (DOC) with agents such as doxorubicin (DOX) is the conventional treatment regime for metastatic and locally advanced BC [37]. A phase III multi-center study [38] compared the efficacies between first-line chemotherapy, DOC and DOX combination (DD), DOX and cyclophosphamide (DC) combination, for the treatment of metastatic BC. However, it is reported that patients suffer from febrile neutropenia and infections after receiving the treatments, especially for those receiving DOC and DOX. Besides, the mitomycin C/methotrexate combination is reported to be effective in metastatic BC patients who have received multiple aggressive treatment regimens [39]. However, this combination regimen resulted in hematological toxicity, hair loss and gastrointestinal toxicity [40]. Some other patients received the combination treatments of DOC and epirubicin (DE), or 5-fluorouracil (5-FU), epirubicin and cyclophosphamide (FEC), which are used as first-line chemotherapy for metastatic BC. However, there is a a significant increase in thrombocytopenia and leukopenia in the patients who received FEC-DG (dose-reduced docetaxel) treatments.

First-line treatment for ER/PR+ (hormone receptor-positive) BC usually develops resistance within 3 months [41], and additional targeted agents were evaluated in numerous clinical trials, such as immune checkpoints, inhibitors of CDK4/6, mTOR and an endocrine therapy combination [42]. The first HER2-targeted drug, trastuzumab, was shown to improve this subtype prognosis [43]; however, resistance is also common in this treatment regimen. Despite having the recent HER2-targeted combination therapies with pertuzumab, lapatinib, neratinib and pertuzumab, trastuzumab and taxane [44,45], the progression-free survival cannot exceed 6 months [46]. Novel chemotherapeutic agents and strategies are needed for the treatments of BC such as DOX and paclitaxel [47]. Although new treatments were used to overcome drug resistance, the median overall survival for basal-like TNBC and non-basal TNBC from metastasis to death is only 6 months and 11 months, respectively [48]. Based on the adverse effects and drug resistance observed in the clinical studies, seeking natural and safe compounds for the treatment of BC is desperately needed. In this review, a potential and safe compound, fucoxanthin, will be introduced. Researchers have been studying this compound in previous years; however, there are no reviews with a particularity of fucoxanthin effects against BC. Here, we will provide an updated summary with a novel aspect to discuss the anti-breast cancer effects of fucoxanthin and its underlying mechanisms of action.

## 2. Fucoxanthin

### 2.1. Sources

Fucoxanthin (Fx) is a xanthophyll derivative found in the chloroplasts of seaweeds and diatoms, which include Heterokontophyta (Chrysophyceae, Raphidophyceae, Bacillariophyceae and Phaeophyceae), Haptophyta, Macrophytic Rhodophyta and Dinophyta [49]. It belongs to the class of non-provitamin A marine carotenoids with a natural brown- or orange-colored pigment [50]. Although it accounts for only around 10% of the estimated total natural production of carotenoids, Fx is the most prevalent of all the carotenoids [51].

### 2.2. Chemical Structures

The chemical structure of Fx comprises an allenic bond, a 5,6-monoepoxide, 9 conjugated double bounds and numerous of functional groups, including hydroxyl, carbonyl and carboxyl groups [52]. The allenic bond is unique to Fx, which makes Fx unstable and becomes heat, aerial and illumination sensitive [53]. Fx exists in either trans or cis configuration. The trans-isomer is a potent antioxidant and relatively stable compared to cis-isomer. The trans-isomer accounts for around 90% of all the Fx found in nature [54,55]. The ability of Fx to quench reactive oxygen and nitrogen species in xanthophylls is similar to carotenes, which contributes to its lipophilicity and antioxidant activities [56]. It is suggested that the high antioxidant activities of Fx is due to the presence of an allenic bond in the acetyl functional group [57,58]. However, the polarity of xanthophylls is higher than carotenes due to the existence of the hydroxyl and epoxide groups [59]. Numerous studies proved that Fx and Fucoxanthinol (Fxol) exert strong anti-inflammatory [60,61], anti-obesity [61,62,63,64], photoprotective [65,66], anti-oxidative [57,67,68,69], neuroprotection [70], antibacterial [71], anti-carcinogenic [72,73] and anti-angiogenic [74] effects both in vitro and in vivo. However, the pharmacological effects of Amarouciaxanthin A, a metabolite of Fx, are not well-studied.

### 2.3. Absorption

Orally administered Fx will be hydrolyzed in the gastrointestinal tract to form Fxol by the digestive enzymes such as cholesterol esterase and lipase, before it is absorbed by the intestinal cells [75,76]. There are several steps for the carotenoid’s absorption in the intestinal area. Carotenoids are first released from the food matrix, which will then solubilize into mixed lipid micelles in the lumen and finally enter into the intestinal mucosal cells [77]. It is believed that carotenoids are absorbed into the enterocyte as scavenger receptor class B type 1 (SR-B1), cluster of differentiation 36 (CD36) and Niemann-Pick C1-like 1 (NPC1L1) by the apical membrane transporters, and SR-BI may be the carrier of Fx in intestinal absorption [78]. It was demonstrated that Fx is esterified in human intestinal Caco-2 cells and combined with lipids to form chylomicrons for the systemic transport [79,80]. Beside Fxol, researchers also identified another metabolite in the plasma and liver after Fx consumption, which is a biotransformed metabolite from Fxol, Amarouciaxanthin A [81]. Amarouciaxanthin A is the oxidative product of Fxol produced in the liver by the liver microsomal NAD-dependent dehydrogenase, which will be rapidly transported to the other tissues, and it mainly accumulates in adipose tissues [82,83]. At the end, amarouciaxanthin A will be metabolized to amarouciaxanthin B [84,85].

### 2.4. Safety

Fx is promoted as a nutrition supplement because it is well-known for its health benefits such as anti-cancer, anti-obesity, anti-diabetic and anti-inflammatory effects [51,86]; besides, it also prevents chronic diseases [80]. Unlink therapeutics, supplements are applicable to the general population. Hence, the safety of Fx being a supplement becomes the major concern. Fortunately, numerous research projects have proved the safety of Fx at various dosages.

The safety of purified Fx was proved by many studies. No mortality, abnormalities or adverse effect are reported in mice treated with a single dose of Fx (1000 and 2000 mg/kg) or repeated doses (500 and 1000 mg/kg) for 30 days [76]. Similarly, no adverse effect is observed in rodents after receiving 200 mg/kg Fx for 3 months [87]. In a human double-blind placebo-controlled study, overweight adults with administration of Fx (1 or 3 mg daily) for 4 weeks had a significantly lower BMI and visceral fat area without abnormal vital clinical signs and parameters [88]. It is interesting to note that continuous intake of Fx will turn the outer color and internal tissues of mice to orange, but no significant toxicity is observed [82]. It is because the selectivity in the intestinal absorption limits the carotenoids’ accumulation in human tissue [89]; therefore, Fx will not accumulate in our body and induce significant toxicity. Based on these results, Fx is suggested to be a safe nutraceutical ingredient and can be further investigated in drug fabrication [90].

## 3. Anti-Breast Cancer Effects of Fucoxanthin

### 3.1. Anti-Proliferative Effect

The anti-proliferation effects of Fx and Fxol in BC cells have been explored by many researchers (Table 1). Numerous studies show that Fx and Fxol significantly reduce the cell viabilities of MCF7, SKBR3 and MDA-MB-231 cells in dose- and time-dependent manners [91,92,93,94,95,96]. Rwigemera reported that Fx and Fxol reduce the cell viability of MDA-MB-231 cells to a greater extent, while Fxol exhibits stronger anti-proliferation effects than Fx does. Fxol is thought to contribute to the reduction in the viability of aggressive estrogen-independent tumor growth by inhibiting nuclear translocation and members of transcriptional activity in the NF-κB signaling pathway [94]. The inhibition of NF-κB may also lead to the reduction in MCF-7 cells’ viability since Fx/Fxol induces apoptosis and reduces the nuclear NF-κB transcription factors p65 and p100 in MCF-7 cells. In addition, Rwigemera believes that Fx and Fxol inhibit the viability of estrogen-resistant BC cells by down-regulating the SOX9 phosphorylation. It is interesting to note that Fx can also reduce cell viability of CMT-U27 cells, which are canine mammary tumor cells, in a dose-dependent manner [97].

Other than cell viability, DNA fragmentation also leads to cell death. Konishi, Funahashi and their colleagues show an increase in DNA fragmentation in BC cells after Fx and Fxol treatments, indicating the anti-proliferative effects of Fx and Fxol. However, another study showed that Fx neither induces detectable cell death nor DNA damage in the BC cells [96]. Different experimental conditions may contribute to the discrepancies, such as the culture conditions, Fx concentrations, treatment protocols and the analytical methods for DNA damage and cell death.

There are also in vivo experiments showing the anti-proliferation effects of Fx/Fxol-enriched extracts. A study used *wakame* seaweed to study the anti-proliferation effects of Fx [101]. *Wakame* (Undaria *pinnatifida*) is usually harvested as a food source, and the sporophyll from *wakame* is often discarded; however, it contains a significant amount of Fx (~20–50% of Fx in the blade part of the *wakame*) [102]. Data proved that cancer-bearing animal models fed with *wakame*-containing diets have reduced tumor growth; in particular, the cancer-bearing rats fed with 5% *wakame* in the diet had almost no tumor growth. Bromodeoxyuridine (BrdU) is a thymidine analog that incorporates to DNA in the cells, which is commonly used as marker to indicate cell proliferation. Mammary tumor-bearing rats fed with *wakame* have low levels of the labeling index (LI) of BrdU in the resected mammary tumors after Fx treatments, suggesting that Fx suppresses tumor growth by inhibiting cancer cell proliferation [101]. In addition, an inverse relationship in LI of BrdU and the apoptotic index (AI) was seen while a positive relationship between the TGF-β and AI is observed. Besides, TGF-β is a paracrine and autocrine hormone that inhibits cancer growth and induces apoptosis in BC cells [103]. Kesari found an inverse relationship between angiogenesis and apoptosis, and the downregulation of angiogenesis was due to the inhibition of endothelial proliferation [104], suggesting that TGF-β is a paracrine growth factor. These results suggest that Fx increases TGF-β expression, induces apoptosis and eventually inhibits tumor cell proliferation. Funahashi also conducted another study on *mekabu* instead of *wakame*. *Mekabu* is one of the brown seaweed species; it contains a considerable amount of Fx and other bioactive organic compounds [98]. The study shows that *mekabu* also exhibits a remarkable inhibitory effect on the cancer growth in vivo [99], which again suggests the anti-cancer effects of Fx.

Another study further demonstrated the anti-proliferative effects of Fx and Fxol in BC [100]. Tumors are composed of a diversified cell population, and their formation and maintenance are rooted from the subpopulation of cells with both stem and cancer cell characteristics [105]. Cancer stem cells (CSCs) have the ability to divide asymmetrically, which means they can further increase the CSCs’ population and undergo differentiation to generate diversified cell types within tumors via self-renewal [106]. Researchers currently suggest that most of the solid tumors, including BC, are stem cell disorders, with stem cells being crucial for dispersion and metastasis [107,108]. CD44^+^CD24^−^, being the representative marker of BC stem cells (BCSCs), allows small cell subpopulations to regenerate the tumor from as little as 100 cells [109]. This phenomenon indicates that only a small number of BCSCs can potentially form tumor spheres or mammospheres [110]. De la Mare demonstrated that despite the incomplete elimination of mammosphere formation after Fx treatments, Fx significantly reduces the sphere forming efficiency (SFE) by ~50%, and the mammosphere size is reduced dose-dependently in BC. Since there is an increase in CD44^+^/CD24^−^ in mammospheres, therefore, the putative anti-CSCs mechanism behind may relate to the inhibition of the signal transduction pathway.

The tumor-specific cytotoxicity of Fx remains controversial since several researchers state that the absence of cytotoxicity is caused by Fx in normal cells [111,112]. Funahashi showed that there is a strong apoptosis induction of *mekabu* in MCF-7, T-47D and MDA-MB-231 cells, but at the same time no apoptotic effect was induced in normal human mammary cells [99]. However, de La Mare and his colleagues found that Fx at 10 μM reduced the viability of MCF12A cells by around 71%. Malhão et al. also found that the viability of MCF12A cells is greatly affected by Fx. Therefore, the tumor-specific cytotoxicity of Fx/Fxol may require further investigation.

### 3.2. Apoptotic Effect

It is suggested that the anticancer effects of Fx and Fxol are mainly due to their apoptotic activities in cancer cells [55]. A study showed that there is a strong apoptosis induction of *mekabu* in the human BC cell lines (MCF-7, T-47D and MDA-MB-231) [99] (Table 2), and the apoptotic effect is even stronger than that of 5-fluorouracil (5-FU), which has been a first-line drug for BC since the 1960s [113].

It is reported that Fxol generally exerts a greater apoptotic effect than Fx does [93,94], while the sensitivity of both treatments in MDA-MB-231 cells is generally higher than that in MCF-7 cells [93,94,99]. Fx and Fxol treatments not just induce apoptosis but also necrosis [94]. However, Rwigemera indicated that there was no significant change in necrosis for both Fx and Fxol treatments [90]. The reasons behind these findings may root from the modulatory actions of Fx and Fxol in the NF-κB singling pathway. The results showed that Fxol inhibits p50, p52/p100, p65 and Rel-B nuclear accumulations in MDA-MB-231 cells, which are all transcription factors in the NF-κB signaling pathway [52], while 10 μM Fxol can consistently reduce phosphorylation of p65 in the nucleus of both MCF-7 and MDA-MB-231 cells. p65 is a marker in metastatic tumors, and it is constituently active in most of the BC subtypes. p65 contributes to the conversion of BC growth to hormonal independence [114]. Phosphorylation of p65 at Ser536 can lead to lymphatic invasion and lymph node metastasis [52], and enhance cell motility, transformation and transcriptional activity [115]. Therefore, apoptotic effects of Fxol in BC cells may be due to the reduction in p65 phosphorylation, and the different apoptotic responses observed in MDA-MB-231 and MCF-7 cells are probably due to the different inhibitory mechanisms involving canonical (p65) and non-canonical (p52, Rel-B) NF-κB signaling pathways.

SOX9 is proved to be a downstream target of many signaling pathways that contributes to BC aggressiveness and is linked to poor clinical outcomes [116]. It is suggested that with the presence of retinoic acid, the nuclear accumulation of SOX9 inhibits BC growth [117,118] while the increase in cytoplasmic accumulation of SOX9 correlates with metastatic BC [119]. Rwigemera found that no nuclear accumulation of SOX9 (correlated to mRNA expression) is observed after Fx/Fxol treatments, suggesting that SOX9 does not inhibit cell growth via nuclear accumulation [93], while a reduction in SOX9 levels in the nucleus is observed at higher doses of Fx or Fxol (20 μM) in MDA-MB-231 cells, suggesting that SOX9 activity may be involved. It is also suggested that AKT directly phosphorylates Sox9 at serine 181 and Sox9 was identified as a novel AKT substrate [120]. Since it was proved that Fx is able to suppress PI3K/Akt/NF-κB signaling [95], therefore, the inhibitory effects on the viability of estrogen-resistant BCs caused by Fx and Fxol may be due to the downregulation of SOX9 phosphorylation [94]. The expression of SOX9 is closely related to SOX10 in TNBC and basal/stem-like BCs [120]. Therefore, future studies can be conducted to explore the roles of the SOX9-SOX10 axis in the anti-BC effects of Fx.

Fx can also induce apoptosis in CMT-U27 cells, which are canine mammary tumor cells, in a dose-dependent manner [97]. Apoptosis is precisely controlled by caspase3, caspase 7, caspase 8 activities. Caspase-8, which induces apoptosis extrinsically together with Fas associated via death domain (FADD) by forming the death-inducing signaling complex (DISC) [121]. PAPR is a family of enzymes involved in many cellular processes such as DNA repair, cell proliferation and cell death [122]. PARP cleavage, which inhibits DNA repair and rehabilitates apoptosis after DNA damage [123], is one of the biomarkers for apoptosis. Therefore, the elevation in caspase 8, cleaved-caspase 8, PARP and cleaved-PARP caused by Fx in CMT-U27 cells suggests an apoptotic effect.

### 3.3. Anti-Metastatic Effects

Cancer metastasis involves cell invasion and migration, angiogenesis and intravasation, survival in the circulation and attachment to the endothelium, extravasation and lastly colonization [124]. Most cancer chemotherapies or drug research mainly focus on cell invasion and migration because, once the cancer cells enter the circulation, they will be developed into stage III or even stage IV cancer [125], and chemotherapy becomes relatively ineffective.

It was proved that Fx is able to reduce the migration and invasion of MDA-MB-231 cells in a dose-dependent manner [95], which may be due to the reduced expressions of VEGF-C. VEGF-C is one of the lymphangiogenic factors that binds to VEGFR-3 that enhances lymphatic vessels to invade tumors [126]. Another study shows that Fx reduces the expressions and secretions of matrix metalloproteinases-2 (MMP-2) and MMP-9 while increasing metalloproteinase-1 (TIMP-1) expression [127]. The anti-inflammatory mechanism of Fx may contribute to its anti-metastatic property since immune cells will migrate and invade to sites of inflammation that involve the degradation of ECM and adjustment of cytokine and chemokine activities [128]. MMPs play a pivotal role in assisting tumor cells’ invasion and migration [129], while the activities of MMPs are specifically adjusted by tissue inhibitors’ TIMPs [128]. In addition, Fx inhibits the migratory ability of CMT-U27 cells and HUVECs in both time- and dose-dependent manners [97]. These results suggest the inhibitory effects of Fx on BC cell migration and invasion.

### 3.4. Anti-Angiogenic Effects

Angiogenesis is the process of recruiting new blood vessels, which is essential in metastasis as it is the principal route to deliver oxygen and nutrients to the tumor cells. The vascular density is associated with the prognostic outcome, and the higher the vascular density in primary tumors, the higher the potential of metastasis [130]. Neovascularization in angiogenesis significantly contributes to BC progression and dissemination. BC cells are able to secrete pro-angiogenic factors, such as fibroblast growth factor (FGF), vascular endothelial growth factor (VEGF), interleukins (ILs), transforming growth factor beta (TGF-β), platelet-derived growth factor (PDGF), that control the angiogenesis and metastasis since they can trigger neovascularization [131].

Due to the high incidence and mortality rate of BCs among the female population, many studies have focused on the angiogenesis in breast tumors. In most of these studies, human umbilical vein endothelial cells (HUVECs) were recognized as the universal endothelial cells model to inspect the antiangiogenic activities of drugs on neovascularization [132]. Sugawara claims that Fx has high antiangiogenic activity in HUVECs due to its ability to suppress tube formation and endothelial cell proliferation but not migration [74]. VEGF receptor-2 is a well-known receptor involved in angiogenic signaling and regulating tumor migration [133]. However, as mentioned, Fx does not affect the migration of HUVECs; therefore, Fx might not affect the VRGF receptor-2 signaling. Other than Fx, Fxol also significantly suppresses the outgrowth of microvessels in a dose-dependent manner [74]. Since Fxol is a metabolite of Fx, therefore, Fx is proposed to be an in vivo bioactive component in suppressing angiogenesis [81].

Jang also reported that Fx possesses anti-angiogenic activity that is due to its ability to reduce the microvascular sprouting of HUVEC by 25% [97]. Moreover, the tubule formation of HUVECs is significantly inhibited after treatments. These results suggest that Fx has an anti-angiogenic effect and prevents the sprouting of new blood vessels. Factors related to angiogenesis include VEGF, EGF, insulin-like growth factor (IGF) and Ang2 [134,135,136,137]. Ang2 inhibits endothelial cell death and vessel regression, and induces migration, proliferation and sprouting in the presence of VEGF, while it will exert opposite effects when VEGF is absent [137]. The anti-angiogenic mechanism of Fx was deciphered in Jang’s research. It was observed that Fx increases the mRNA level of Ang2 in both HUVEC and CMT-U27 cells while the levels of VEGF-A and VEGFR-2 remained unchanged. Furthermore, Fx is shown to reduce the protein expression of VE-cadherin, which is a component located at junctions to determine the vascular integrity of the endothelial cell [138], meaning that Fx is able to weaken the cell-to-cell junction.

Wang used human lymphatic endothelial cells (HLEC) as a lymphangiogenesis model and determined the inhibitory effects of Fx [95]. The results show that Fx inhibits tube formation and migration of HLEC by suppressing PI3K/Akt/NF-κB signaling. The signaling targets in this pathway are reported to mediate tumor proliferation, metastasis, angiogenesis, migration and adhesion, and the degradation of the ECM [112]. Therefore, inhibition of the PI3K/Akt/NF-κB signaling pathway induced by Fx can inhibit angiogenesis. Indeed, as mentioned by Rwigemera, Fx affects the protein expression of both canonical and non-canonical pathways in the NF-κB signaling cascade. Other than p50, p52, p65, p100 and RelB, IκB and IKK are also involved in this signaling pathway. It is reported that a NF-κB-induced lncRNA acts as tumor suppressor to inhibit BC metastasis by inhibiting the phosphorylation of IκB induced by IKK but without affecting the activity of IKK [139]. Therefore, the inhibition in NF-κB signaling may greatly contribute to the antiangiogenic effect of Fx. Beside the in vitro studies, Fx also inhibits tumor-induced lymphangiogenesis in both a HLEC and MDA-MB-231 BC xenograft model by reducing micro-lymphatic vascular density [95]. The inhibition of lymph node metastasis in BC caused by Fx may be due to the inhibition of MMP-2 and MMP-9 secretion and elevation of TIMP-1 expression [95]. Since lymphangiogenesis is associated with lymph node metastasis in the presence of VEGF-C that is secreted by MDA-MB-231 cells [140], therefore, the downregulation of the VEGF-C and VEGFR3 signaling axis contributes to the anti-lymphangiogenesis activity. Other than the potential targets stated above, some studies suggest the association between the antiangiogenic effect and the antioxidant activity of Fx since reactive oxygen species (ROS) stimulate angiogenesis [141,142].

### 3.5. Modulation of Tumor Microenvironment

Tissue-resident macrophages are intrinsic immune cells possessing phagocytic activities under physiological conditions. They play an essential role in tissue homeostasis maintenance and pathogen defense due to their heterogeneous characteristics with tissue- and niche-specific functions [143]. The TME in BC includes immune system elements such as macrophages, neutrophils, lymphocytes and dendritic cells, cells composing blood vessel, fibroblast, myofibroblast, mesenchymal stem cells, adipocytes and ECM [144,145]. The most protruding TME member in these cells is the tumor-associated macrophages (TAMs), which mediate tumor proliferation by secreting growth factors and inflammatory mediators such as CCL2, IL-1α, IL-6 and TNF-α [146] and induce treatment resistance in cancer [147]. Notably, TNF-α released by TAMs contributes to the activation of NF-κB in tumor cells, thus preventing tumor cell death and promoting tumor cell invasion [148]. The anti-inflammatory cytokines produced by TAMs recruit Treg cells, which are able to suppress the activation of the effector T cell and eventually suppress the immune response in TME [149]. TAM-derived chemokines, such as IL-4, IL-10, TGF-β and prostaglandin-E2 (PGE2), can directly suppress the functions of cytotoxic T cells [150,151].

Within the breast tumor, TAMs may comprise over half of the cell numbers. The accumulated TAMs in BC are composed of resident macrophages (RMs) and monocytes recruited from the circulation [152]. The monocyte colony stimulating factor will then turn RMs into non-polarized (M0) macrophages [153]. M0 macrophages have high plasticity as they can be transformed into different phenotypes with environmental stimulations. Macrophages can exist as two unique phenotypes after polarization, which are the classically activated (M1) or the alternative activated (M2) macrophages. In human BC, the high density of TAMs is associated with poor clinical prognosis [154]. Over the past few decades, TAMs were reported to have the ability to remodel the tumor ECM to assist invasion, induce angiogenesis, shape BC cells to escape from the host immune system and recruit immunosuppressive leukocytes to the TME [145].

M1 macrophages can be induced by proinflammatory factors, such as TNF-α, lipopolysaccharide (LPS) and cytokines, through the granulocyte–macrophage colony-stimulating factor. After that, interleukins (IL) -1β, IL-6, ROS and nitric oxide (NO) are released to promote tumor proliferation [149] and at the same time induce the polarized Th1 response. Th1 response is a proinflammatory response which will trigger the Th2 response when it is in excess [155]. Here, a feedback loop is formed, since the Th2 response will release more interleukins and further enhance the proinflammatory effects, eventually leading to tumorogenesis. M2 macrophages, which are activated by Th2-related cytokines (IL-13, IL-4), or other related signals, such as IL-10, glucocorticoid hormones and TGF-β, have the ability to scavenge molecules and produce suppressive mediators, such as polyamines and mannose or galactose receptors [156,157]. M2 macrophages usually facilitate canonical tissue repair functions under normal physiology. However, they can also be pro-carcinogenic by promoting tissue remodeling and repair, stimulating angiogenesis with VEGF and enhancing tissue proliferation with TGF-β [149]. Therefore, controlling the levels of inflammatory mediators in BC is extremely important.

As mentioned above, pro-inflammatory mediators such as NO, PGE_2_, TNF-α, IL-1β and IL-6 promote tumorogenesis. However, there is less study focused on the association between Fx and the TME in BC. Nevertheless, the anti-inflammatory effects of Fx isolated from Ishige *okamurae* in lipopolysaccharide (LPS)-stimulated murine macrophage RAW 264.7 cells are proved [158]. The RAW 264.7 cells are monocyte/macrophage-like cells, which are an authoritative model of macrophages commonly used to investigate the anti-metastatic effects of treatments [159] and that can demonstrate pinocytosis and phagocytosis. Fuentes proved that RAW 264.7 cells stimulated by LPS have a higher NO production and phagocytosis rate [160]. Kim’s study demonstrated the anti-inflammatory mechanism of Fx [158]. Fx reduces pro-inflammatory mediators such as NO, PGE_2_, IL-1β, TNF-α, and IL-6 by inhibiting NF-κB activity, cytoplasmic degradation of inhibitors of IκB-α and nuclear translocation of p50 and p65 proteins and MAPK (JNK, ERK and p38) phosphorylation in RAW 264.7 cells. However, further investigations are needed to confirm the effects of Fx in BC.

### 3.6. Modulation of Carcinogen Metabolism

Cytochrome P450 (CYP) is a xenobiotic metabolizing enzyme. CYP1A1, CYP1A2 and CYP3A4 are reported to contribute to the pro-carcinogenic activities, and their expressions are significantly affected by Fx [161]. In the genetic polymorphisms of human cytochromes’ P450 enzymes, a correlation between CYP1A1 and CYP1C2, which is used to increase activity of 17β-estradiol and estrone, is observed that will increase BC risk [162]. A pharmacogenetic study also pointed out the association between the CYP2A6 genotype and the plasma letrozole concentration in postmenopausal women with BC, which may serve as a predictor [163].

CYP enzymes are membrane-bound hemoproteins which can synthesize second messengers, hormones and other endogenous substances in the body, detoxify xenobiotics and regulate cellular metabolism [164,165]. CYP1A1 activates Benzo[a]pyrene (B[a]P) and other carcinogenic polycyclic aromatic hydrocarbons (PAHs); CYP1A2 catalyzes metabolic activation of aryl-, heterocyclic amine and PAH-diols to reactive metabolites; CYP3A4 metabolizes the endogenous compound and therapeutic drugs and activates mycotoxins [166]. CYPs are widely expressed in organs under normal conditions [167] to catalyze drug molecules for second-phase metabolism and excretion [168] while CYPs are selectively expressed in different types of neoplasms under BC [169]. Recently, Luo proposed the association between CYP enzymes and tumorigenesis [170].

CYPs contribute to the risk and prognosis of BC due to their participance in estrogen metabolism. CYP3A4 is shown to be negatively associated with the morbidity of BC [171]. Additionally, it is reported that the morbidity of BC patients with late menarche is negatively associated with the CYP3A polymorphism site rs10235235 [172]; women with an age below 50 who have a non-coding variant at the CYP3A locus (rs10273424) usually have lower risk of developing BC [173]. A genetic study in Thailand revealed that CYP1A2, CYP2C19 and CYP17 polymorphisms play an essential role in estrogen metabolism and may increase the BC risk [174]. Furthermore, Bai suggests CYP1A2 rs2470890 to be a genetic indicator of BC prognosis due to its prominent association with the BC prognostic rate [175]. Therefore, the expression and activities of CYPs greatly contribute to the BC risk.

The high prediction value of Fx (0.76 in CYP3A4) in silico results indicate that Fx has an inhibitory effect on the metabolic enzymes that are engaged in carcinogen metabolism [176,177]. The enzymatic activities of CYP1A2 and CYP3A4 are inhibited by Fx in a dose-dependent manner, and the IC_50_ values reach 30.3 μM and 24.4 μM, respectively. Molecular docking results further proved the inhibitory effect of Fx by comparing the binding activities to the known inhibitors α-naphthoflavone and ketoconazole. The binding energy of Fx for CYP1A2 and CYP3A4 are −4.83 kcal mol^−1^ and −7.69 kcal mol^−1^, respectively [176]. These data demonstrate that Fx is a preventive compound and potential anti-carcinogenic agent which inhibits the metabolizing enzyme activities.

CYPs are essential for carcinogens’ metabolism [178], and their enzymatic activities will affect the susceptivity to chemical carcinogens in human [179]. CYPs activate polycyclic aromatic hydrocarbons (PAHs), which are common environmental carcinogens, to induce tumorigenesis [180]. PAHs will accumulate in breast tissues [181] and cause mutation after they are metabolized and activated by CYP1A1 [182]. Therefore, it is important to know the significance of CYPs in contributing to the initiation of BC. The aryl hydrocarbon receptor (AhR) is a transcriptional regulator of CYP1A1, and it is reported that the AhR/CYP1A1 signaling pathway contributes to the tumor development and chemoresistance of BCSCs by inhibiting the tensin homolog and phosphatase and activating β-catenin and Akt signaling pathways [183]. Therefore, future cancer studies can investigate the relationship between AhR/CYP1A1 and Fx in ER-negative BC. CYPs’ induction-mediated interaction is also well-known in reducing therapeutic efficacy [184]; thus, inhibition of CYPs may help to overcome multidrug resistance (MDR) in BC.

### 3.7. Overcome Multidrug Resistance

MDR is a clinical impediment observed in over 80% of patients with all kinds of cancer chemotherapy. The reduced drug efficacy caused by MDR may eventually lead to a high dosage that results in high toxicity and also financial burden for the patients [185]. To overcome MDR, seeking a novel ATP-Binding Cassette transporter (ABCT) inhibitor is pivotal. Studies have identified 48 genes in human encoding of the ABCT transporter superfamily, which are classified into seven subgroups (A to G) phylogenetically [186]. There is an intricate system in ABC transporters responsible for physiological functions, including passive diffusion that regulates the intracellular levels of ions, lipids, hormones, xenobiotics and other small molecules [187,188,189] and regulation of organelles, such as the mitochondrion, lysosome, endoplasmic reticulum and Golgi apparatus to preserve the physiological homeostasis [189]. Multidrug resistance protein 1 (MDR1), MDR-associated protein 1 (MRP1) and BC resistance protein (BCRP) are well-known ABCTs that promote drug efflux against the concentration gradient and reduce cellular accumulation, thus inducing MDR by allowing cancer cells to escape from the pharmacological barriers [190,191,192,193].

Fx is currently being studied for its synergistic interaction with front line drugs to overcome MDR [194]. Fx was reported to reduce the adverse effects of ROS-stimulating cytotoxic drugs in normal cells while enhancing the cytotoxicity in cancer cells due to the antioxidant characteristics. The synergistic effect found in Fx combination treatments suggests the potential of Fx to become a drug adjuvant in cancer treatment [195,196]. Indeed, it was reported that treatment with a minimal cytotoxic concentration of DOX with the physiological dose of Fx significantly reduces the cell viability of MCF-7 and MDA-MB-231 cells by 68% and 53%, respectively [197]. In addition, the IC_50_ values for MCF-7 and MDA-MB-231 cells are significantly reduced by nearly five times in Fx and DOX combination treatment when compared to Fx monotreatment [197]. These results demonstrated the synergistic effect of Fx in combination with DOX in inhibiting BC cell viability. Malhão also suggests Fx to be a potential drug adjuvant based on the cytotoxic results in 2D- and 3D-cultured BC cell models such as MCF7, SKBR3 and MDA-MB-231 cells with Fx alone or combined with Dox and cisplatin (Cis) [96]. Malhão also claims that the synergistic effects of this combination are more pronounced in the TNBC cells [96]. In order to reveal the reversal effects of Fx in MDR, a study used adriamycin (DOX) resistance cell lines MCF-7/ADR to examine the effects of Fx in overcoming drug resistance [90]. The results show that the cytotoxic effect of Fx is weakened in both parent cells and resistant cells, and the resistance cell line is insensitive to Fx or DOX monotreatment. However, DOX and Fx combination treatment can remarkably lower the IC_50_ value of DOX in MCF-7/ADR cells, suggesting the reversal effect of this combination treatment in BC.

Fx is also reported to have synergistic inhibitory effects on BC cell proliferation. Ki67, a prognostic marker for BC [198], is expressed in all phases of the cell cycle except the G0 phase [96,199]. In a 3D culture study, the antiproliferative effect of Fx is nonsignificant; however, the combination of Fx 20 μM with Dox 1 μM significantly reduces BC cell proliferation by 50%, which exhibits a similar effect to Dox (5 μM) treatment [96]. These results suggest the potential adjuvant ability of Fx in augmenting the antiproliferative effect of Dox.

The Fx and DOX combination treatment also induces apoptosis in MCF-7/ADR cells in which the early apoptosis induced by the combination treatment is at least double of that in the Fx or DOX monotreatment [90]. Apoptosis can be used to assess the cellular response to chemotherapy [200], and the commonly used biomarker is cleaved caspase-3 [201], which is one of the cysteine proteases and plays an essential role in apoptotic pathways by cleaving cellular proteins [202]. The Fx and DOX combination treatment increases the expressions of apoptotic genes, including CASP3, CASP8 and P53, and reduces the expressions of metabolic genes CYP3A4 (phase I metabolism), GST (phase II metabolism) and PXR. Besides, the treatment also reduces the expressions of transporter genes ABCC1, ABCG2 and ABCB1 when compared to Fx or DOX monotreatment [90]. Similarly, the study from Malhão showed that the expression of caspase-3 in MDA-MB-23 cells treated with 20 μM Fx alone is similar to the control group. However, the combination of 20 μM Fx and 2 μM Dox significantly increases the expression of caspase-3 positive cells and is similar to 5 μM Dox monotreatment. The significant increase in apoptosis and expression of cleaved caspase 3 reinforce the synergistic effects Fx and Dox combination treatment and suggest that Fx is a potent compound that can be used with other first line drugs to overcome MDR in BC.

In Eid’s study, Fx (20 μM) significantly enhances the accumulation of DOX in MCF-7/ADR cells, and the effect is even stronger than verapamil (known inhibitor of ABCT) [90]. A similar result is also found when comparing the inhibitory effect of Fx for Rho123 (a fluorescent ABCT substrate) accumulation [90]. The relative resistance value showed that Fx is a good substrate for P-gp-expressing cells. Taken together, these results suggest that Fx is an ABCT substrate. Therefore, the cytotoxicity of Fx may be indirectly due to the ABCT competitive efflux. Generally, BCRP and MRP1 are co-expressed with ABCTs’ P-gp/MDR1, and their substrates and inhibitors are common. Thus, Fx probably induces a synergistic effect by affecting the activity of P-gp/MDR1, BCRP and MRP1 in BC cells.

Besides, the reason underlying 25% of ER-positive breast tumor patients developing NF-κB antagonists’ resistance is due to the constitutive expression and activation of NF-κB members [94], which will eventually lead to estrogen-independent growth [203,204,205]. The constitutive nuclear localization of p50, p52, c-Rel and over-expression of p100/p52 are found in BC [206]. Besides, p65 is activated in most human BC cell lines and correlated with more aggressive and metastatic BC [94]. Therefore, the inhibitory effects of Fxol on p65, p52 and Rel-B nuclear accumulations found in MDA-MB-231 cells can help to overcome the MDR induced by the overexpression of NF-κB members. Indeed, numerous studies reported the association between the p65 phosphorylation and chemoresistance in response to DOX [207,208,209]. It is suggested that IKKα, which is an upstream kinase that can modulate p65 phosphorylation levels, plays a critical role in NF-κB-mediated chemoresistance in response to DOX, and it potentially serves as a therapeutic target for improving the chemotherapeutic response [117]. Therefore, Fx may sensitize DOX and overcome drug resistance by regulating the phosphorylation of p65.

Both Vijay and Malhão showed that the Fx and Dox combination treatment has higher cytotoxicity to MDA-MB-231 cells than the monotreatments. The promising dosages of the combination treatment are Fx at 10 μM and Dox at 1 μM, which exert potent anti-cancer effects [96,197]. It is generally believed that 3D cell cultures are more resistant to drug treatments and better translate organism-level realities [210,211]. The use of 3D cell cultures in Malhão’s study also suggest that Fx is a latent drug adjuvant. Other than the above-mentioned mechanisms, the synergistic cytotoxicity mechanisms may comprise of other complex systems, such as DNA damage, cell cycle arrest and ROS induction. Rwigemera emphasized the involvement of the NF-κB pathway in the development of BC resistance and that Fx can target this pathway to overcome MDR [93,94]. In conclusion, the above studies strongly suggest that Fx is a potential drug adjuvant; however, more in vitro and in vivo studies are needed to probe the underlying mechanisms of the synergistic anti-BC effect of the combination of Fx and Dox.

### 3.8. Anti-Oxidative Effects and Cancer Prevention

ROS are a family comprised of molecules that have an unpaired electron in their atomic orbital and can exist independently [212]. ROS include free radicals namely superoxide anion (O_2_^−^), hydrogen peroxide (H_2_O_2_), hydroxyl radical (OH), organic hydroperoxide (ROOH), peroxy radicals (ROO) and hypochlorous acid (HOCl) [212,213,214]. Singlet oxygen (^1^O_2_) and free radicals are produced by the aerobic metabolism in the body [215]. These oxidants can react with proteins, DNA or lipids to induce damage or structural changes, leading to mutation, transformation and eventually carcinogenesis. Luckily, the antioxidation effects of seaweeds are broadly investigated [216], and Fx consists of the dietary antioxidants found to have the ability to enhance the antioxidant capacity of blood serum levels in mammals.

A study shows that Fx exerts significant antioxidant activity in MCF-7 cells in the ABTS experiment and was proved to have protective activity to DNA damage [92]. Many researchers also proved the tremendous singlet oxygen quenching and radical scavenging activity of Fx/Fxol [57,69,217,218]. Beside in vitro studies, an ex vivo study also demonstrates the antioxidant activity of Fx extracted from Fucus *vesiculosus* [219].

The antioxidant effect of Fx/Fxol may depend on the source and structure. Interesting data suggested the antioxidant activities of microalgal extracts and Fx/Fxol, in which the Fx content in fresh *wakame* is ~50% more than in processed *wakame* (drying), while the fresh *wakame* showed a significant reduction in the DPPH radical scavenging and CUPRAC assays [96]. The research from Kawee-ai proved the ability of Fx to donate an electron, and its reducing ability increased in a dose-dependent manner [68]. The stabilization and termination of the radical chain induced by the reactions between free radicals and reductones explain the reducing ability of Fx. It is noteworthy that the IC_50_ value of the microalgal Fx (0.30 mM) is almost double that of Fx extracted from brown seaweed (164.6 μM), suggesting that microalgal Fx may have a potent therapeutic effect. Sachindra also demonstrated the scavenging and quenching ability of Fx and Fxol in most types of oxidants such as DPPH, ABTS, hydroxyl, superoxide radical and singlet oxygen [57], suggesting the antioxidant activities of Fx and Fxol. The scavenging activities of Fx and Fxol were linearly dependent on the concentrations, and both of them showed a similar effective concentration. It was reported that the presence of functional groups in the terminal rings, such as carbonyl and hydroxyl groups, reduces the ABTS scavenging activity of carotenoids [220]. However, Fxol has three hydroxyl groups compared to two in Fx, and Fxol exhibits higher ABTS scavenging activity than Fx. It may be due to the existence of the acetyl group in Fx. Therefore, the effects of functional groups to antioxidant activities need to be further investigated. However, it is certain that the allenic bond is responsible for the high antioxidation effects of Fx and Fxol. Sachindra also proposed that the presence of conjugated keto groups can increase the quenching rate while the presence of a hydroxyl, epoxy and methoxy group will lower the effect [57]. Fx and its two metabolites have a conjugated keto group, but their effects are still weaker than β-carotene. The reduction in signal intensity of hydroperoxide in the presence of Fx is mainly due to scavenging of the radical directly, but at the same time, the possibility was proposed that the carotenoid interferes with the enzyme system [57].

It is notable that Kawee-ai revealed the association between the ratio of the cis- and trans-isomer of Fx and its antioxidation effects, in which the higher the ratio of cis-isomer, the lower the antioxidant activity. Other than configurations, it is suggested that the polarity and lipophilicity of carotenoids will also affect the antioxidant activities [69]. Besides, the extraordinary characteristic of Fx is that it does not donate a proton to ROS similar to other antioxidants, but an electron [51], and that Fx is able to quench ROS under hypoxia [217,218].

Other than the structural effect of carotenoids, the nature of the radical may also affect the scavenging activity [221]. It is reported that the pigments in carotenoids are responsible for the quenching ability by acting as catalysts to inactivate the ^1^O_2_. The process starts by transferring the electron exchange energy from ^1^O_2_ and the carotenoid to generate the triplet state of the carotenoid (^3^CAR) and ground state oxygen (^3^O_2_). Then, with the rotational and vibrational interactions under a solvent system, the ^3^CAR formed will eventually return to a ground state by dissipating its energy through [67]. Rodrigues also summarizes the mechanisms of how carotenoids scavenge ROO and OH, which include electron transfer, abstraction of the allylic hydrogen and radical addition to the conjugated double bonds system [69].

Despite the anti-oxidative effects, the role of ROS remains controversial in cancer because it can either enhance or inhibit tumorigenesis under different concentrations [157]. The MAPK/ NF-κB pathway will be stimulated under moderate ROS levels, which will upregulate the expression of MMPs and VEGF, and thus lead to cancer proliferation, angiogenesis and metastasis [222]. However, proapoptotic proteins Bax, p21 and p27 will be activated under high intracellular ROS levels while the antiapoptotic Bcl-2 and Bcl-xL will be suppressed [195]. Therefore, it is interesting to explore the roles of ROS in mediating the anti-BC effects of Fx.

### 3.9. Anti-Obesity Effect and Cancer Prevention

The World Health Organization estimates that 40% of adult women are overweight, with the prevalence tripling between 1975 and 2016 [223]. There has been numerous research with BMI data suggesting that central obesity is a risk factor of BC [224,225]. DeSantis also suggests that the increasing prevalence of overweight underlies the increased HR-positive BC cases in USA [2]. The comorbidity of obesity is reported to be a risk factor of BC in postmenopausal women by affecting the estrogen receptor signaling, such as excessive local production of estrogens in adipose tissues, production of adipokines and inflammatory cytokines and hypercholesterolemia [226,227].

Obesity contributes to premenopausal and postmenopausal BC risk in different ways [228]. It was reported that high BMI is negatively associated with premenopausal BC risk while the opposite trend is observed in postmenopausal women [229]. Some studies claimed that there is a positive relationship between obesity and risk of BC in premenopausal ER-negative and TNBC since the ER−/PR− tumor is more common in obese women compared to the ER+/PR+ tumor [230], while other studies suggest that BMI is inversely associated with premenopausal ER+ BC [230,231]. Studies suggest that obese postmenopausal women may have a higher risk of suffering from hormone receptor-positive BC [231,232,233]. The EPIC cohort studies also suggest that obesity is associated with more advances BC in postmenopausal women [234]. Therefore, it seems that obesity is closely related to ER−/PR− BC in premenopausal women while it is related to ER+/PR+ BC in postmenopausal women [231]. Obesity not just increases the risk of having BC but also increases BC mortality. Higher BMI is reported to have poorer BC-specific survival compared to normal weight BC women [235,236]. Therefore, preventing and reducing obesity are the keys to reduce BC cases.

Fx is also well-known for its anti-obesity properties. Its underlying mechanisms have been linked to the upregulation of UCP 1, which is at the center of brown adipose tissue (BAT) thermogenesis and systemic energy homeostasis, helping to reduce fat accumulation [237,238,239,240,241]. Indeed, the expression of UCP1 in white adipose tissue (WAT) was significantly enhanced while the WAT weight was significantly reduced after Fx treatment in mice [62]. It is believed that the UCP1 expressed in WAT generates heat and causes energy dissipation and eventually leads to weight loss. It is reported that PPARα, PPARγ, iNOS and COX-2 are able to modulate the expression of UCP1 in WAT [242,243,244,245], and their expressions can be regulated by Fx [239,245,246]. Therefore, the regulatory effect of Fx in UCP1-mediated thermogenesis underlies its anti-obesity effect.

Studies also reveal the metabolism effects of Fx. Fx can effectively reduce liver triglyceride and total cholesterol levels and enhance the excretions of these lipids by fecal samples in rats [247]. The expressions of lipogenic enzymes ACC, FAS, and G6PDH and the transcriptional factor of SREBP-1c are significantly reduced while the expression of lipid-metabolizing enzymes CPT1 and CYP7A1 are significantly increased after Fx treatments [247]. Abidov also reported that Fx significantly reduces body weight, body and liver fat content and serum triglycerides (TG) [248]. Besides, Jeon reported that Fx exerts anti-obesity effects by reducing the activities of the enzymes involved in fatty acid (FA) synthesis, FA oxidation and TG synthesis in both liver and epididymal adipose tissue. Fx also reduces the activities of enzymes involved in cholesterol biosynthesis and esterification such as the hepatic HMG-CoA reductase and acyl coenzyme A [249]. Malhão observed a lower electron density of the lipid droplets in multicellular aggregates exposed to Fx [96]. Since electron density of lipid droplets reflects fatty acid composition [250], therefore, it is again suggesting the regulatory effects of Fx on fatty acid synthesis and lipid metabolism. Overall, Fx exerts anti-obesity effects by regulating the plasma and hepatic lipid profiles, fecal lipids, fatty acid synthesis, lipid absorption and hepatic cholesterol metabolism.

As mentioned above, obesity is closely associated with BC. Therefore, the anti-obesity effects of Fx may help to prevent BC to a certain extent although there are no relevant clinical data. However, different studies already proved the ability of carotenoids in preventing BC. High intake of carotenoids can lower the risk of having BC by nearly 20% when compared to low intake [251,252]. The Nurses’ Health Study also reported that a mean intake of fruit and vegetables above 5.5 servings/day significantly reduces BC risk by 11%. Therefore, being the most potent carotenoid, Fx is believed to have the ability to prevent BC by reducing obesity.

The anti-obesity mechanism of Fx is complicated. Other than UCP1 and the branch metabolism effects induced by Fx, studies also suggest the relationship between obesity and the antioxidative/antiangiogenic effects of Fx [247,253,254]. Since Fx exerts both antioxidative and antiangiogenic activities, the obesity preventive and reductive mechanism behind Fx can be intricate.

## 4. Conclusions and Future Perspective

**Figure 1:**
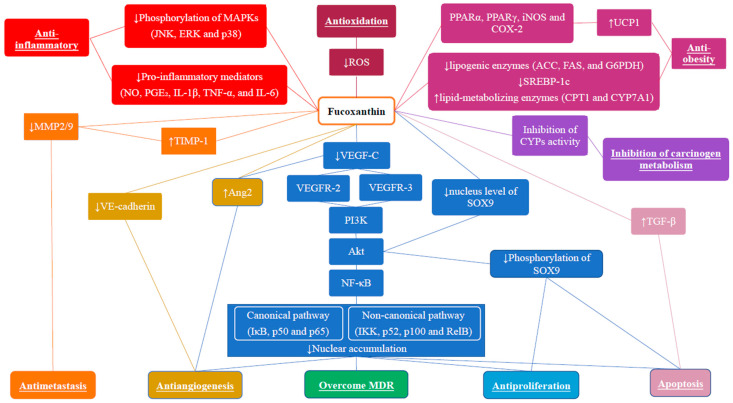
Anticancer and cancer prevention effects of Fucoxanthin (Fx) and Fucoxanthinol (Fxol). The most important molecular pathways involved in Fx/Fxol’s mechanisms of action are also depicted.

Figure 1 summarizes the anticancer effects of Fx and Fxol, which include suppression of cancer proliferation, metastasis, angiogenesis and carcinogen metabolism, induction of apoptosis, scavenging and quenching of free radicals and regulation of TME. Since the involvement of NF-κB members is frequently observed, it is believed that the NF-κB pathway is the key to contributing to the anti-cancer mechanism of Fx. The anti-obesity and anti-oxidative effects of Fx and Fxol potentially contribute to BC prevention; however, further investigation is needed. Besides, Fx and Fxol are believed to be potential adjuvant drugs for BC chemotherapy in overcoming resistance to first-line drugs and augmenting their efficacies. It is believed that more in-depth studies on Fx and Fxol will bring novel and exciting therapeutic strategies for the treatment of BC.

Fx is currently gaining great attention due to its potent anti-proliferative effects in many cancer types. However, insufficient clinical evidence and lack of a global picture delineating the mechanisms of action underlying its therapeutic effects have hindered its clinical application in BC treatment. Therefore, it is essential to increase the number of research investigations of Fx for BC in both in vivo and in vitro designs. Only by raising the novelty of Fx can the implication of Fx in humans be made possible.

In fact, Fx is proved to inhibit angiogenesis in HUVECs and HLECs and regulate TME in RAW 264.7 cells. It is worth investigating its anti-angiogenic effects in BC cell models. Besides, 3D cultured cell models that imitate a realistic cancer situation can also be used. Furthermore, research related to the anti-obesity effects and the regulation of CYP enzymes’ activity also has huge implications, and such research may act as the pioneer to the metabolism study of Fx in BC.

Other than the anti-cancer effects, Fx is also proved to be a potential drug adjuvant in BC. Despite the advancement of neoadjuvant chemotherapy, the development of drug resistance remains the biggest challenge in treating BC. The use of combination therapy is a common therapeutic regimen for BC patients since it can increase the efficacy of the treatments and at the same time prevent tolerance in tumor cells. Therefore, including Fx for BC combination therapy may provide more options to the clinics and benefit more patients in the future.

The safety and anti-cancer effects of Fx and Fxol have already proved by many animal experiments. Clinical trials will be the next to validate the efficacy and safety of Fx and Fxol in BC treatment. Beside the clinical use, Fx is also believed to be a good dietary supplement due to its low cytotoxicity, high nutrient value and remarkable cancer multi-prevention effects. The cost-effective factor of Fx makes the bulk production worth considering since abundant sporophyll can be harvested from the seaweeds.

## Figures and Tables

**Table 1:** Antiproliferative effects of Fucoxanthin (Fx), Fucoxanthinol (Fxol) and Fx/Fxol-rich extract in breast cancer cell lines.

Algal Extract or Compound	Cell Type(s)	Study Design	Anti-Proliferation Effects	Reference
Organic extract of *Halocynthia roretzi*, Fx	MCF-7	Fx/Fxol were dissolved in ethanol adjusted to less than 0.5% in volume. Viable MCF-7 cell number was measured colorimetrically with WST-1 reagent.	Cell viability: ~90% after 48 h at 25 μM ~60% after 72 h at 25 μM	[91]
Organic extract of *Halocynthia roretzi*, Fxol	MCF-7		Cell viability: ~30% after 48 h at 25 μM ~15% after 72 h at 25 μM	
Organic extract of *Halocynthia roretzi*, Fx	MCF-7	Fx/Fxol were dissolved in ethanol adjusted to less than 0.5% in the culture medium. The DNA fragments were stained with ethidium bromide and visualized.	DNA fragmentation level: 2-fold of ctrl after 48 h at 12.5 μM 6-fold of ctrl after 48 h at 25 μM	[91]
Organic extract of *Halocynthia roretzi*, Fxol	MCF-7		DNA fragmentation level: 7-fold of ctrl after 48 h at 12.5 μM 12-fold of ctrl after 48 h at 25 μM	
Methanol extract of *Sargassum*, Fucoxanthin (60 mg, 0.017% dry wt.)	MCF-7	The viability of the cells was examined by microscopical examination using hemocytometer and trypan blue stain.	IC_50_ = 11.5 μM Cell viability: ~60% after 24 h at 20 μM ~30% after 48 h at 20 μM	[92]
Methanol extract of *Sargassum*, Fucoxanthin (60 mg, 0.017% dry wt.)	MCF-7	Bleomycin-dependent DNA damage assay with absorbance measured at 532 nm.	DNA fragmentation level: 39-fold of ctrl after 24h at 20 μM 42-fold of ctrl after 48h at 20 μM	[92]
Fx	MCF-7	Treatments of BC cells with 20, 30, or 40 μM of Fx/Fxol in a time-dependent (12, 24, or 48 h) manner, cell-titer blue cell viability assay was done, the amount of reduced resorufin was measured as fluorescence signal at 560Ex/590Em	IC_50_ = 121.89 μM Cell viability: ~70% after 24h at 30 μM ~50% after 24 h at 40 μM ~20% after 48 h at 30 μM ~10% after 48 h at 40 μM	[93]
	MDA-MB-237		IC_50_ = 141.54 μMCell viability: ~50% after 24 h at 30 μM~30% after 24 h at 40 μM~20% after 48h at 30 μM~10% after 48 h at 40 μM	
Fxol	MCF-7		IC_50_ = 39.63 μM Cell viability: ~60% after 12 h at 40 μM ~40% after 24 h at 40 μM ~10% after 48h at 40 μM	
	MDA-MB-237		IC_50_ = 33.59 μM Cell viability: ~40% after 12 h at 40 μM ~20% after 24 h at 40 μM >10% after 48 h at 40 μM	
Fx	MCF-7	Treatments of BC cells with 10 and 20 μM of Fx/Fxol in a time-dependent (6, 12, 24 or 48 h) manner, cell-Titer blue cell viability assay was done, the amount of reduced resorufin was measured by its fluorescence signal at 560Ex/590Em	Non-significant	[94]
	MDA-MB-237		Cell viability: ~90% after 48 h at 20 μM	
Fxol	MCF-7		Cell viability: ~40% after 48 h at 20 μM	
	MDA-MB-237		Cell viability: ~80% after 24 h at 20 μM ~50% after 48 h at 20 μM	
Fx	MDA-MB-237	Treatments of MDA-MB-231 cells with Fx (25, 50, 100 μmol/L) for 12, 24 or 48 h. MTT assay with absorbance was measured at 492 nm.	Cell viability: ~90% after 24 h at 100 μM ~70% after 48 h at 100 μM	[95]
Fx	MDA-MB-231 xenograft model	Five days after BC cell inoculation, Fx (100, 500 μmol/L; 100 μL/mouse) was injected into the tumour peripheral every day for 26 days. Tumour size was measured every 4 days.	Tumor volume: 100 μmol/L group is ~20% smaller after 26 days 500 μmol/L group is ~60% smaller after 26 days	[95]
			Tumor weight: 100 μmol/L group is ~30% smaller after 26 days 500 μmol/L group is ~60% smaller after 26 days	
Fx	MCF-7	Treatments of BC cells with 10, 20 and 50 μM of Fx. MTT assay was done with absorbance measured at 570 nm.	Cell viability: ~70% after 72 h at 10 μM ~40% after 72 h at 20 μM ~20% after 72 h at 50 μM	[96]
	SKBR3		Cell viability: ~80% after 72 h at 10 μM ~40% after 72 h at 20 μM ~10% after 72 h at 50 μM	
	MDA-MB-237		Cell viability: ~70% after 72 h at 10 μM ~20% after 72 h at 20 μM ~10% after 72 h at 50 μM	
Fx	CMT-U27	Treatments of BC cells with 0, 5, 10, and 20 μM of Fx. Crystal violet staining with absorbance was measured at 550 nm.	Cell viability: ~60% after 24 h at 10 μM ~45% after 24 h at 20 μM	[97]
*Wakame*	Female Sprague-Dawley (SD) rats	Rats in control group (I-A) and group (I-B and I-C) were given *wakame* seaweed of 1.0% and 5.0% of their body weight, respectively, for 8 weeks. Changes in the body weight and tumor size were measured.	Tumor size: I-B is ~60% smaller in week 8 I-C is ~80% smaller in week 8 and no significant increase in tumor size since week 0	[98]
*Mekabu,* ~6.7 mg/mL	MCF7	1.0 g of powdered *mekabu* was dissolved in 150 mL of distilled water and 1 mL of *mekabu* solution was added to culture medium. DNA fragmentation was analyzed by apoptosis ladder detection kit.	DNA fragmentation level: 2.5-fold of ctrl after 96 h	[99]
	T-47D		DNA fragmentation level: 2-fold of ctrl after 96 h	
*Mekabu* solution	Female Sprague-Dawley (SD) rats	Powdered *mekabu* 1.5 g was mixed with 1000 mL of distilled water and was filtered as *mekabu* solution. Weekly changes in body weight, incidence and the number of mammary tumors in each rat were observed for 32 weeks.	Tumor size of *mekabu* group is ~95% smaller after 32 weeks	[99]
Fx	MCF-7	Treatments of MCF-7 cells with Fx (0, 5, 10, 15 μM) and growth of the mammospheres for 8 days. WST-1 assay was done with absorbance measured at 450 nm.	Cell viability: ~90% after 24 h at 5 μM ~80% after 48 h at 10 μM	[100]

**Table 2:** Apoptotic effects of Fucoxanthin (Fx), Fucoxanthinol (Fxol) and Fx/Fxol-rich extract in breast cancer cell lines.

Algal Extract or Compound	Cell Type(s)	Study Design	Apoptosis %	Reference
Fx	MCF-7	Treatments of BC cells with 20 μM Fx/Fxol followed by staining with FITC-Annexin V, ethidium homodimer III and Hoechst 33342. Fluorescence was assessed using an Axio Observer A1 inverted fluorescence microscope with FITC, rhodamine and DAPI filters	<10% after 12 h at 20 μM	[93]
	MDA-MB-231		~50% after 12 h at 20 μM	
Fxol	MCF-7		~40% after 12 h at 20 μM	
	MDA-MB-231		~40% after 12 h at 20 μM	
Fx	MCF-7	Treatments of BC cells with 10 μM Fx/Fxol followed by staining with FITC-Annexin V, ethidium homodimer III and Hoechst 33342. Fluorescence was assessed using an Axio Observer A1 inverted fluorescence microscope with FITC, rhodamine and DAPI filters	~40% after 24 h at 10 μM	[94]
	MDA-MB-231		~60% after 24 h at 10 μM	
Fxol	MCF-7		~70% after 24 h at 10 μM	
	MDA-MB-231		~60% after 24 h at 10 μM	
Fx	CMT-U27	Treatments of BC cells with 20 μM Fx. Flow cytometric annexin V assay was used, and fluorescence was measured at 488 nm (excitation) and 525 nm (emission)	~60% after 24 h at 20 μM	[97]
*mekabu*	MCF7	Treatments of BC cells with 1.0 g *mekabu* solution followed by staining with propidium iodide (PI). Fluorescence Intensity for PI was measured by flow cytometry gated by FSC vs. SSC.	~30% after 24 h at 15 μM ~60% after 72 h at 15 μM	[99]
	MDA-MB-237		~70% after 24 h at 15 μM ~70% in 72 h at 15 μM	
	T-47D		~60% after 24 h at 15 μM ~70% after 72 h at 15 μM	

## Data Availability

Data sharing not applicable.

## References

[1] Siegel R.L., Miller K.D., Jemal A. (2020). Cancer statistics, 2020. CA Cancer J. Clin..

[2] DeSantis C.E., Ma J., Gaudet M.M., Newman L.A., Miller K.D., Goding Sauer A., Jemal A., Siegel R.L. (2019). Breast cancer statistics, 2019. CA Cancer J. Clin..

[3] Iacoviello L., Bonaccio M., de Gaetano G., Donati M.B. (2021). Epidemiology of breast cancer, a paradigm of the “common soil” hypothesis. Semin. Cancer Biol..

[4] World Health Organization (WHO) Estimated number of New Cases from 2020 to 2040, Both Sexes, Age [0–85+]. https://gco.iarc.fr/tomorrow/en/dataviz/isotype?cancers=20&single_unit=100000&types=0.

[5] World Health Organization (WHO) Estimated Number of Deaths from 2020 to 2040, Both Sexes, Age [0–85+]. https://gco.iarc.fr/tomorrow/en/dataviz/isotype?cancers=20&single_unit=100000&types=1.

[6] Sun Y.S., Zhao Z., Yang Z.N., Xu F., Lu H.J., Zhu Z.Y., Shi W., Jiang J., Yao P.P., Zhu H.P. (2017). Risk Factors and Preventions of Breast Cancer. Int. J. Biol. Sci..

[7] Allison K.H. (2012). Molecular Pathology of Breast Cancer. Am. J. Clin. Pathol..

[8] Prat A., Perou C.M. (2010). Deconstructing the molecular portraits of breast cancer. Mol. Oncol..

[9] Prat A., Ellis M.J., Perou C.M. (2011). Practical implications of gene-expression-based assays for breast oncologists. Nat. Rev. Clin. Oncol..

[10] Lehmann B.D., Bauer J.A., Chen X., Sanders M.E., Chakravarthy A.B., Shyr Y., Pietenpol J.A. (2011). Identification of human triple-negative breast cancer subtypes and preclinical models for selection of targeted therapies. J. Clin. Investig..

[11] Dittmer J. (2018). Breast cancer stem cells: Features, key drivers and treatment options. Semin. Cancer Biol..

[12] Sotiriou C., Neo S.-Y., McShane L.M., Korn E.L., Long P.M., Jazaeri A., Martiat P., Fox S.B., Harris A.L., Liu E.T. (2003). Breast cancer classification and prognosis based on gene expression profiles from a population-based study. Proc. Natl. Acad. Sci. USA.

[13] Balmaña J., Diez O., Rubio I.T., Cardoso F. (2011). BRCA in breast cancer: ESMO Clinical Practice Guidelines. Ann. Oncol..

[14] Paluch-Shimon S., Cardoso F., Sessa C., Balmaña J., Cardoso M.J., Gilbert F., Senkus E. (2016). Prevention and screening in BRCA mutation carriers and other breast/ovarian hereditary cancer syndromes: ESMO Clinical Practice Guidelines for cancer prevention and screening. Ann. Oncol..

[15] Foulkes W.D. (2003). Germline BRCA1 Mutations and a Basal Epithelial Phenotype in Breast Cancer. Cancer Spectrum Knowl. Environ..

[16] Turner N.C., Reis-Filho J.S. (2006). Basal-like breast cancer and the BRCA1 phenotype. Oncogene.

[17] Turner N.C., Reis-Filho J.S., Russell A.M., Springall R.J., Ryder K., Steele D., Savage K., Gillett C.E., Schmitt F.C., Ashworth A. (2006). BRCA1 dysfunction in sporadic basal-like breast cancer. Oncogene.

[18] Carey L.A., Perou C.M., Livasy C.A., Dressler L.G., Cowan D., Conway K., Karaca G., Troester M.A., Tse C.K., Edmiston S. (2006). Race, Breast Cancer Subtypes, and Survival in the Carolina Breast Cancer Study. JAMA.

[19] Morris G.J., Naidu S., Topham A.K., Guiles F., Xu Y., McCue P., Schwartz G.F., Park P.K., Rosenberg A.L., Brill K. (2007). Differences in breast carcinoma characteristics in newly diagnosed African–American and Caucasian patients. Cancer.

[20] Bane A.L., Beck J.C., Bleiweiss I., Buys S.S., Catalano E., Daly M.B., Giles G., Godwin A.K., Hibshoosh H., Hopper J.L. (2007). BRCA2 Mutation-associated Breast Cancers Exhibit a Distinguishing Phenotype Based on Morphology and Molecular Profiles from Tissue Microarrays. Am. J. Surg. Pathol..

[21] Deng C.-X. (2006). BRCA1: Cell cycle checkpoint, genetic instability, DNA damage response and cancer evolution. Nucleic Acids Res..

[22] Dine J., Deng C.-X. (2012). Mouse models of BRCA1 and their application to breast cancer research. Cancer Metastasis Rev..

[23] Davis N.M., Sokolosky M., Stadelman K., Abrams S.L., Libra M., Candido S., Nicoletti F., Polesel J., Maestro R., D’Assoro A. (2014). Deregulation of the EGFR/PI3K/PTEN/Akt/mTORC1 pathway in breast cancer: Possibilities for therapeutic intervention. Oncotarget.

[24] Elizalde P.V., Russo R.I.C., Chervo M.F., Schillaci R. (2016). ErbB-2 nuclear function in breast cancer growth, metastasis and resistance to therapy. Endocr. Relat. Cancer.

[25] Appert-Collin A., Hubert P., Crémel G., Bennasroune A. (2015). Role of ErbB Receptors in Cancer Cell Migration and Invasion. Front. Pharmacol..

[26] Ali R., Wendt M.K. (2017). The paradoxical functions of EGFR during breast cancer progression. Signal Transduct. Target. Ther..

[27] Zhang D., LaFortune T.A., Krishnamurthy S., Esteva F., Cristofanilli M., Liu P., Lucci A., Singh B., Hung M.-C., Hortobagyi G.N. (2009). Epidermal Growth Factor Receptor Tyrosine Kinase Inhibitor Reverses Mesenchymal to Epithelial Phenotype and Inhibits Metastasis in Inflammatory Breast Cancer. Clin. Cancer Res..

[28] Alanazi I.O., Khan Z. (2016). Understanding EGFR Signaling in Breast Cancer and Breast Cancer Stem Cells: Overexpression and Therapeutic Implications. Asian Pac. J. Cancer Prev..

[29] Kim A., Jang M.H., Lee S.J., Bae Y.K. (2017). Mutations of the Epidermal Growth Factor Receptor Gene in Triple-Negative Breast Cancer. J. Breast Cancer.

[30] Xu J., Chen Y., Olopade O.I. (2010). MYC and Breast Cancer. Genes Cancer.

[31] Chen Y., Olopade O.I. (2008). MYC in breast tumor progression. Expert Rev. Anticancer Ther..

[32] Jung M., Russell A.J., Liu B., George J., Liu P.Y., Liu T., DeFazio A., Bowtell D., Oberthuer A., London W.B. (2016). A Myc Activity Signature Predicts Poor Clinical Outcomes in Myc-Associated Cancers. Cancer Res..

[33] Fernandez-Medarde A., Santos E. (2011). Ras in Cancer and Developmental Diseases. Genes Cancer.

[34] Ray A., Ray B.K. (2014). Induction of Ras by SAF -1/ MAZ through a feed-forward loop promotes angiogenesis in breast cancer. Cancer Med..

[35] Saeidi S., Kim S.-J., Han H.-J., Kim S.H., Zheng J., Lee H.-B., Han W., Noh D.-Y., Na H.-K., Surh Y.-J. (2020). H-Ras induces Nrf2-Pin1 interaction: Implications for breast cancer progression. Toxicol. Appl. Pharmacol..

[36] Banys-Paluchowski M., Milde-Langosch K., Fehm T., Witzel I., Oliveira-Ferrer L., Schmalfeldt B., Müller V. (2019). Clinical relevance of H-RAS, K-RAS, and N-RAS mRNA expression in primary breast cancer patients. Breast Cancer Res. Treat..

[37] Esteva F.J., Valero V., Pusztai L., Boehnke-Michaud L., Buzdar A.U., Hortobagyi G.N. (2001). Chemotherapy of Metastatic Breast Cancer: What to Expect in 2001 and beyond. Oncologist.

[38] Fisusi F.A., Akala E.O. (2019). Drug Combinations in Breast Cancer Therapy. Pharm. Nanotechnol..

[39] Tanabe M. (2016). Combination Chemotherapy of Mitomycin C and Methotrexate Was Effective on Metastatic Breast Cancer Resistant to Eribulin, Vinorelbine, and Bevacizumab after Anthracycline, Taxane, and Capecitabine. Case Rep. Oncol..

[40] Panasci L., Shenouda G., Begin L., Pollak M., Reinke A., Margolese R. (1990). Mitomycin C and mitoxantrone chemotherapy for advanced breast cancer: Efficacy with minimal gastrointestinal toxicity and alopecia. Cancer Chemother. Pharmacol..

[41] Finn R.S., Martin M., Rugo H.S., Jones S., Im S.A., Gelmon K., Harbeck N., Lipatov O.N., Walshe J.M., Moulder S. (2016). Palbociclib and Letrozole in Advanced Breast Cancer. N. Engl. J. Med..

[42] Pernas S., Tolaney S.M., Winer E.P., Goel S. (2018). CDK4/6 inhibition in breast cancer: Current practice and future directions. Ther. Adv. Med. Oncol..

[43] Seidman A.D., Fornier M.N., Esteva F.J., Tan L., Kaptain S., Bach A., Panageas K.S., Arroyo C., Valero V., Currie V. (2001). Weekly Trastuzumab and Paclitaxel Therapy for Metastatic Breast Cancer with Analysis of Efficacy by *HER2* Immunophenotype and Gene Amplification. J. Clin. Oncol..

[44] Blackwell K.L., Burstein H.J., Storniolo A.M., Rugo H.S., Sledge G., Aktan G., Ellis C., Florance A., Vukelja S., Bischoff J. (2012). Overall Survival Benefit with Lapatinib in Combination with Trastuzumab for Patients with Human Epidermal Growth Factor Receptor 2–Positive Metastatic Breast Cancer: Final Results from the EGF104900 Study. J. Clin. Oncol..

[45] Saura C., Garcia-Saenz J.A., Xu B., Harb W., Moroose R., Pluard T., Cortés J., Kiger C., Germa C., Wang K. (2014). Safety and Efficacy of Neratinib in Combination with Capecitabine in Patients with Metastatic Human Epidermal Growth Factor Receptor 2–Positive Breast Cancer. J. Clin. Oncol..

[46] Baselga J., Cortés J., Kim S.-B., Im S.-A., Hegg R., Im Y.-H., Roman L., Pedrini J.L., Pienkowski T., Knott A. (2012). Pertuzumab plus Trastuzumab plus Docetaxel for Metastatic Breast Cancer. N. Engl. J. Med..

[47] Ngamcherdtrakul W., Yantasee W. (2019). siRNA therapeutics for breast cancer: Recent efforts in targeting metastasis, drug resistance, and immune evasion. Transl. Res..

[48] Kennecke H., Yerushalmi R., Woods R., Cheang M.C.U., Voduc D., Speers C.H., Nielsen T.O., Gelmon K. (2010). Metastatic Behavior of Breast Cancer Subtypes. J. Clin. Oncol..

[49] Takaichi S. (2011). Carotenoids in Algae: Distributions, Biosyntheses and Functions. Mar. Drugs.

[50] Martin L.J. (2015). Fucoxanthin and Its Metabolite Fucoxanthinol in Cancer Prevention and Treatment. Mar. Drugs.

[51] Abu-Ghannam N., Shannon E., Gupta V.K., Treichel H., Shapaval V.O., de Oliveira L.A., Tuohy M.G. (2017). Seaweed carotenoid, fucoxanthin, as functional food. Microbial Functional Foods and Nutraceuticals.

[52] Martin M., Hartley A.V., Jin J., Sun M., Lu T., Mozsik G. (2019). Phosphorylation of NF-κB in cancer. Adenosine Triphosphate in Health and Disease.

[53] Zhang H., Tang Y., Zhang Y., Zhang S., Qu J., Wang X., Kong R., Han C., Liu Z. (2015). Fucoxanthin: A Promising Medicinal and Nutritional Ingredient. Evid.-Based Complement. Altern. Med..

[54] Holdt S.L., Kraan S. (2011). Bioactive compounds in seaweed: Functional food applications and legislation. J. Appl. Phycol..

[55] Nakazawa Y., Sashima T., Hosokawa M., Miyashita K. (2009). Comparative evaluation of growth inhibitory effect of stereoisomers of fucoxanthin in human cancer cell lines. J. Funct. Foods.

[56] Kim S., Chojnacka K. (2015). Marine Algae Extracts: Processes, Products, and Applications.

[57] Sachindra N.M., Sato E., Maeda H., Hosokawa M., Niwano Y., Kohno M., Miyashita K. (2007). Radical Scavenging and Singlet Oxygen Quenching Activity of Marine Carotenoid Fucoxanthin and Its Metabolites. J. Agric. Food Chem..

[58] Sangeetha R.K., Bhaskar N., Baskaran V. (2009). Comparative effects of β-carotene and fucoxanthin on retinol deficiency induced oxidative stress in rats. Mol. Cell. Biochem..

[59] Landrum J.T. (2010). Carotenoids: Physical, Chemical, and Biological Functions and Properties.

[60] Dai Y.L., Jiang Y.F., Lu Y.A., Yu J.B., Kang M.C., Jeon Y.J. (2021). Fucoxanthin-rich fraction from *Sargassum fusiformis* alleviates particulate matter-induced inflammation in vitro and in vivo. Toxicol. Rep..

[61] Grasa-López A., Miliar-García Á., Quevedo-Corona L., Paniagua-Castro N., Escalona-Cardoso G., Reyes-Maldonado E., Jaramillo-Flores M.-E. (2016). *Undaria pinnatifida* and Fucoxanthin Ameliorate Lipogenesis and Markers of Both Inflammation and Cardiovascular Dysfunction in an Animal Model of Diet-Induced Obesity. Mar. Drugs.

[62] Maeda H., Hosokawa M., Sashima T., Miyashita K. (2007). Dietary Combination of Fucoxanthin and Fish Oil Attenuates the Weight Gain of White Adipose Tissue and Decreases Blood Glucose in Obese/Diabetic KK-*A^y^* Mice. J. Agric. Food Chem..

[63] Mikami N., Hosokawa M., Miyashita K., Sohma H., Ito Y.M., Kokai Y. (2017). Reduction of HbA1c levels by fucoxanthin-enriched akamoku oil possibly involves the thrifty allele of uncoupling protein 1 (*UCP1*): A randomised controlled trial in normal-weight and obese Japanese adults. J. Nutr. Sci..

[64] Ojulari O.V., Lee S.G., Nam J.-O. (2020). Therapeutic Effect of Seaweed Derived Xanthophyl Carotenoid on Obesity Management; Overview of the Last Decade. Int. J. Mol. Sci..

[65] Liu Y., Liu M., Zhang X., Chen Q., Chen H., Sun L., Liu G. (2016). Protective Effect of Fucoxanthin Isolated from *Laminaria japonica* against Visible Light-Induced Retinal Damage Both in Vitro and in Vivo. J. Agric. Food Chem..

[66] Chen S.-J., Lee C.-J., Lin T.-B., Peng H.-Y., Liu H.-J., Chen Y.-S., Tseng K.-W. (2019). Protective Effects of Fucoxanthin on Ultraviolet B-Induced Corneal Denervation and Inflammatory Pain in a Rat Model. Mar. Drugs.

[67] Krinsky N.I., Johnson E.J. (2005). Carotenoid actions and their relation to health and disease. Mol. Asp. Med..

[68] Kawee-Ai A., Kuntiya A., Kim S.M. (2013). Anticholinesterase and Antioxidant Activities of Fucoxanthin Purified from the Microalga Phaeodactylum Tricornutum. Nat. Prod. Commun..

[69] Rodrigues E., Mariutti L.R.B., Mercadante A.Z. (2012). Scavenging Capacity of Marine Carotenoids against Reactive Oxygen and Nitrogen Species in a Membrane-Mimicking System. Mar. Drugs.

[70] Zhang L., Wang H., Fan Y., Gao Y., Li X., Hu Z., Ding K., Wang Y., Wang X. (2017). Fucoxanthin provides neuroprotection in models of traumatic brain injury via the Nrf2-ARE and Nrf2-autophagy pathways. Sci. Rep..

[71] Karpiński T.M., Adamczak A. (2019). Fucoxanthin—An Antibacterial Carotenoid. Antioxidants.

[72] Nishino H., Murakoshi M., Tokuda H., Satomi Y. (2009). Cancer prevention by carotenoids. Arch. Biochem. Biophys..

[73] Le Goff M., Le Ferrec E., Mayer C., Mimouni V., Lagadic-Gossmann D., Schoefs B., Ulmann L. (2019). Microalgal carotenoids and phytosterols regulate biochemical mechanisms involved in human health and disease prevention. Biochimie.

[74] Sugawara T., Matsubara K., Akagi R., Mori M., Hirata T. (2006). Antiangiogenic Activity of Brown Algae Fucoxanthin and Its Deacetylated Product, Fucoxanthinol. J. Agric. Food Chem..

[75] Sugawara T., Baskaran V., Tsuzuki W., Nagao A. (2002). Brown Algae Fucoxanthin Is Hydrolyzed to Fucoxanthinol during Absorption by Caco-2 Human Intestinal Cells and Mice. J. Nutr..

[76] Beppu F., Niwano Y., Sato E., Kohno M., Tsukui T., Hosokawa M., Miyashita K. (2009). In vitro and in vivo evaluation of mutagenicity of fucoxanthin (FX) and its metabolite fucoxanthinol (FXOH). J. Toxicol. Sci..

[77] Harrison E.H. (2012). Mechanisms involved in the intestinal absorption of dietary vitamin A and provitamin A carotenoids. Biochim. Biophys. Acta BBA Mol. Cell Biol. Lipids.

[78] During A., Dawson H.D., Harrison E.H. (2005). Carotenoid Transport Is Decreased and Expression of the Lipid Transporters SR-BI, NPC1L1, and ABCA1 Is Downregulated in Caco-2 Cells Treated with Ezetimibe. J. Nutr..

[79] Sugawara T., Yamashita K., Asai A., Nagao A., Shiraishi T., Imai I., Hirata T. (2009). Esterification of xanthophylls by human intestinal Caco-2 cells. Arch. Biochem. Biophys..

[80] Bae M., Kim M.B., Park Y.K., Lee J.Y. (2020). Health benefits of fucoxanthin in the prevention of chronic diseases. Biochim. Biophys. Acta BBA Mol. Cell Biol. Lipids.

[81] Asai A., Sugawara T., Ono H., Nagao A. (2004). Biotransformation of Fucoxanthinol into Amarouciaxanthin A in Mice and HEPG2 cells: Formation and Cytotoxicity of Fucoxanthin Metabolites. Drug Metab. Dispos..

[82] Hashimoto T., Ozaki Y., Mizuno M., Yoshida M., Nishitani Y., Azuma T., Komoto A., Maoka T., Tanino Y., Kanazawa K. (2011). Pharmacokinetics of fucoxanthinol in human plasma after the oral administration of kombu extract. Br. J. Nutr..

[83] Yonekura L., Kobayashi M., Terasaki M., Nagao A. (2010). Keto-Carotenoids Are the Major Metabolites of Dietary Lutein and Fucoxanthin in Mouse Tissues. J. Nutr..

[84] Matsuno T., Ookubo M., Komori T. (1985). Carotenoids of Tunicates, III. The Structural Elucidation of Two New Marine Carotenoids, Amarouciaxanthin A and B. J. Nat. Prod..

[85] Yamano Y., Chary M.V., Wada A. (2013). Stereocontrolled First Total Syntheses of Amarouciaxanthin A and B. Org. Lett..

[86] Jeukendrup A.E., Randell R. (2011). Fat burners: Nutrition supplements that increase fat metabolism. Obes. Rev..

[87] Iio K., Okada Y., Ishikura M. (2011). Single and 13-Week Oral Toxicity Study of Fucoxanthin Oil from Microalgae in Rats. Food Hyg. Saf. Sci..

[88] Hitoe S., Shimoda H. (2017). Seaweed Fucoxanthin Supplementation Improves Obesity Parameters in Mild Obese Japanese Subjects. Funct. Foods Health Dis..

[89] Kotake-Nara E., Nagao A., Sugawara T., Maoka T. (2021). Absorption and Metabolism of Xanthophylls. Marine Carotenoids.

[90] Eid S.Y., Althubiti M.A., Abdallah M.E., Wink M., El-Readi M.Z. (2020). The carotenoid fucoxanthin can sensitize multidrug resistant cancer cells to doxorubicin via induction of apoptosis, inhibition of multidrug resistance proteins and metabolic enzymes. Phytomedicine.

[91] Konishi I., Hosokawa M., Sashima T., Kobayashi H., Miyashita K. (2006). Halocynthiaxanthin and fucoxanthinol isolated from Halocynthia roretzi induce apoptosis in human leukemia, breast and colon cancer cells. Comp. Biochem. Physiol. Part C Toxicol. Pharmacol..

[92] Ayyad S.E., Basaif S., Badria A., Ezmirly S., Alarif W., Badria F. (2011). Antioxidant, cytotoxic, antitumor, and protective DNA damage metabolites from the red sea brown alga Sargassum sp. Pharmacogn. Res..

[93] Rwigemera A., Mamelona J., Martin L.J. (2014). Inhibitory effects of fucoxanthinol on the viability of human breast cancer cell lines MCF-7 and MDA-MB-231 are correlated with modulation of the NF-κB pathway. Cell Biol. Toxicol..

[94] Rwigemera A., Mamelona J., Martin L.J. (2015). Comparative effects between fucoxanthinol and its precursor fucoxanthin on viability and apoptosis of breast cancer cell lines MCF-7 and MDA-MB-231. Anticancer Res..

[95] Wang J., Ma Y., Yang J., Jin L., Gao Z., Xue L., Hou L., Sui L., Liu J., Zou X. (2019). Fucoxanthin inhibits tumour-related lymphangiogenesis and growth of breast cancer. J. Cell. Mol. Med..

[96] Malhão F., Macedo A., Costa C., Rocha E., Ramos A. (2021). Fucoxanthin Holds Potential to Become a Drug Adjuvant in Breast Cancer Treatment: Evidence from 2D and 3D Cell Cultures. Molecules.

[97] Jang H., Choi J., Park J.-K., Won G., Seol J.W. (2021). Fucoxanthin Exerts Anti-Tumor Activity on Canine Mammary Tumor Cells via Tumor Cell Apoptosis Induction and Angiogenesis Inhibition. Animals.

[98] Tanemura Y., Yamanaka-Okumura H., Sakuma M., Nii Y., Taketani Y., Takeda E. (2014). Effects of the intake of Undaria pinnatifida (Wakame) and its sporophylls (Mekabu) on postprandial glucose and insulin metabolism. J. Med. Investig..

[99] Funahashi H., Imai T., Mase T., Sekiya M., Yokoi K., Hayashi H., Shibata A., Hayashi T., Nishikawa M., Suda N. (2001). Seaweed Prevents Breast Cancer?. Jpn. J. Cancer Res..

[100] De la Mare J.-A., Sterrenberg J.N., Sukhthankar M.G., Chiwakata M.T., Beukes D.R., Blatch G.L., Edkins A.L. (2013). Assessment of potential anti-cancer stem cell activity of marine algal compounds using an in vitro mammosphere assay. Cancer Cell Int..

[101] Funahashi H., Imai T., Tanaka Y., Tsukamura K., Hayakawa Y., Kikumori T., Mase T., Itoh T., Nishikawa M., Hayashi H. (1999). Wakame Seaweed Suppresses the Proliferation of 7,12-Dimethylbenz(a)-anthracene-induced Mammary Tumors in Rats. Jpn. J. Cancer Res..

[102] Fung A., Hamid N., Lu J. (2013). Fucoxanthin content and antioxidant properties of *Undaria pinnatifida*. Food Chem..

[103] Knabbe C., Lippman M.E., Wakefield L.M., Flanders K.C., Kasid A., Derynck R., Dickson R.B. (1987). Evidence that transforming growth factor-β is a hormonally regulated negative growth factor in human breast cancer cells. Cell.

[104] Kesari A.L., Chellam V.G., Mathew B.S., Nair M.K., Pillai M.R. (1999). Transforming growth factor beta related to extent of tumor angiogenesis but not apoptosis or proliferation in breast carcinoma. Breast Cancer.

[105] Visvader J.E. (2011). Cells of origin in cancer. Nature.

[106] Lawson J.C., Blatch G.L., Edkins A.L. (2009). Cancer stem cells in breast cancer and metastasis. Breast Cancer Res. Treat..

[107] Peitzsch C., Tyutyunnykova A., Pantel K., Dubrovska A. (2017). Cancer stem cells: The root of tumor recurrence and metastases. Semin. Cancer Biol..

[108] Chang J.C. (2016). Cancer stem cells. Medicine.

[109] Al-Hajj M., Wicha M.S., Benito-Hernandez A., Morrison S.J., Clarke M.F. (2003). Prospective identification of tumorigenic breast cancer cells. Proc. Natl. Acad. Sci. USA.

[110] Ponti D., Costa A., Zaffaroni N., Pratesi G., Petrangolini G., Coradini D., Pilotti S., Pierotti M.A., Daidone M.G. (2005). Isolation and in vitro Propagation of Tumorigenic Breast Cancer Cells with Stem/Progenitor Cell Properties. Cancer Res..

[111] Ishikawa C., Tafuku S., Kadekaru T., Sawada S., Tomita M., Okudaira T., Nakazato T., Toda T., Uchihara J.-N., Taira N. (2008). Antiadult T-cell leukemia effects of brown algae fucoxanthin and its deacetylated product, fucoxanthinol. Int. J. Cancer.

[112] Yamamoto K., Ishikawa C., Katano H., Yasumoto T., Mori N. (2011). Fucoxanthin and its deacetylated product, fucoxanthinol, induce apoptosis of primary effusion lymphomas. Cancer Lett..

[113] Cameron D.A., Gabra H., Leonard R.C. (1994). Continuous 5-fluorouracil in the treatment of breast cancer. Br. J. Cancer.

[114] Nakshatri H., Bhat-Nakshatri P., Martin D.A., Goulet R.J., Sledge G.W. (1997). Constitutive activation of NF-kappaB during progression of breast cancer to hormone-independent growth. Mol. Cell. Biol..

[115] Viatour P., Merville M.P., Bours V., Chariot A. (2005). Phosphorylation of NF-κB and IκB proteins: Implications in cancer and inflammation. Trends Biochem. Sci..

[116] Jana S., Krishna B.M., Singhal J., Horne D., Awasthi S., Salgia R., Singhal S.S. (2020). SOX9: The master regulator of cell fate in breast cancer. Biochem. Pharmacol..

[117] Afonja O., Raaka B.M., Huang A., Das S., Zhao X., Helmer E., Juste D., Samuels H.H. (2002). RAR agonists stimulate SOX9 gene expression in breast cancer cell lines: Evidence for a role in retinoid-mediated growth inhibition. Oncogene.

[118] Chakravarty G., Rider B., Mondal D. (2011). Cytoplasmic compartmentalization of SOX9 abrogates the growth arrest response of breast cancer cells that can be rescued by trichostatin A treatment. Cancer Biol. Ther..

[119] Chakravarty G., Moroz K., Makridakis N.M., Lloyd S.A., Galvez S.E., Canavello P.R., Lacey M.R., Agrawal K., Mondal D. (2011). Prognostic significance of cytoplasmic SOX9 in invasive ductal carcinoma and metastatic breast cancer. Exp. Biol. Med..

[120] Al-Zahrani K.N., Abou-Hamad J., Pascoal J., Labrèche C., Garland B., Sabourin L.A. (2021). AKT-mediated phosphorylation of Sox9 induces Sox10 transcription in a murine model of HER2-positive breast cancer. Breast Cancer Res..

[121] Tummers B., Green D.R. (2017). Caspase-8: Regulating life and death. Immunol. Rev..

[122] Morales J., Li L., Fattah F.J., Dong Y., Bey E.A., Patel M., Gao J., Boothman D.A. (2014). Review of Poly (ADP-ribose) Polymerase (PARP) Mechanisms of Action and Rationale for Targeting in Cancer and Other Diseases. Crit. Rev. Eukaryot. Gene Expr..

[123] Meza-Sosa K.F., Miao R., Navarro F., Zhang Z., Zhang Y., Hu J.J., Hartford C.C.R., Li X.L., Pedraza-Alva G., Pérez-Martínez L. (2022). SPARCLE, a p53-induced lncRNA, controls apoptosis after genotoxic stress by promoting PARP-1 cleavage. Mol. Cell.

[124] Hapach L.A., Mosier J.A., Wang W., Reinhart-King C.A. (2019). Engineered models to parse apart the metastatic cascade. npj Precis. Oncol..

[125] Cancer.Net Stages of Cancer. https://www.cancer.net/navigating-cancer-care/diagnosing-cancer/stages-cancer.

[126] Yoshimatsu Y., Miyazaki H., Watabe T. (2016). Roles of signaling and transcriptional networks in pathological lymphangiogenesis. Adv. Drug Deliv. Rev..

[127] Wang P., Liu Z., Liu X., Teng H., Zhang C., Hou L., Zou X. (2014). Anti-Metastasis Effect of Fucoidan from Undaria pinnatifida Sporophylls in Mouse Hepatocarcinoma Hca-F Cells. PLoS ONE.

[128] Lin S.-W., Gao Z.-X., Lin L.-R., Luo X., Liu L.-L., Yang T.-C. (2019). Treponema pallidum enhances human monocyte migration and invasion by dysregulating the MMP/TIMP balance. Int. Immunopharmacol..

[129] Guo W., Gao X., Zhan R., Zhao Z., Xu K., Tang B. (2021). Tricolor imaging of MMPs to investigate the promoting roles of inflammation on invasion and migration of tumor cells. Talanta.

[130] Zetter B.R. (1998). Angiogenesis and Tumor Metastasis. Annu. Rev. Med..

[131] Badodekar N., Sharma A., Patil V., Telang G., Sharma R., Patil S., Vyas N., Somasundaram I. (2021). Angiogenesis induction in breast cancer: A paracrine paradigm. Cell Biochem. Funct..

[132] Wilkus K., Brodaczewska K., Kajdasz A., Kieda C. (2021). Distinctive Properties of Endothelial Cells from Tumor and Normal Tissue in Human Breast Cancer. Int. J. Mol. Sci..

[133] Shibuya M., Claesson-Welsh L. (2006). Signal transduction by VEGF receptors in regulation of angiogenesis and lymphangiogenesis. Exp. Cell Res..

[134] Carmeliet P. (2005). VEGF as a Key Mediator of Angiogenesis in Cancer. Oncology.

[135] Dobrucki L.W., Tsutsumi Y., Kalinowski L., Dean J., Gavin M., Sen S., Mendizabal M., Sinusas A.J., Aikawa R. (2010). Analysis of angiogenesis induced by local IGF-1 expression after myocardial infarction using microSPECT-CT imaging. J. Mol. Cell. Cardiol..

[136] Fagiani E., Christofori G. (2013). Angiopoietins in angiogenesis. Cancer Lett..

[137] Lobov I.B., Brooks P.C., Lang R.A. (2002). Angiopoietin-2 displays VEGF-dependent modulation of capillary structure and endothelial cell survival in vivo. Proc. Natl. Acad. Sci. USA.

[138] Giannotta M., Trani M., Dejana E. (2013). VE-Cadherin and Endothelial Adherens Junctions: Active Guardians of Vascular Integrity. Dev. Cell.

[139] Liu B., Sun L., Liu Q., Gong C., Yao Y., Lv X., Lin L., Yao H., Su F., Li D. (2015). A Cytoplasmic NF-κB Interacting Long Noncoding RNA Blocks IκB Phosphorylation and Suppresses Breast Cancer Metastasis. Cancer Cell.

[140] Sun L., Duan J., Jiang Y., Wang L., Huang N., Lin L., Liao Y., Liao W. (2015). Metastasis-associated in colon cancer-1 upregulates vascular endothelial growth factor-C/D to promote lymphangiogenesis in human gastric cancer. Cancer Lett..

[141] Shono T., Ono M., Izumi H., Jimi S.I., Matsushima K., Okamoto T., Kohno K., Kuwano M. (1996). Involvement of the transcription factor NF-kappaB in tubular morphogenesis of human microvascular endothelial cells by oxidative stress. Mol. Cell. Biol..

[142] Yasuda M., Ohzeki Y., Shimizu S., Naito S., Ohtsuru A., Yamamoto T., Kuroiwa Y. (1998). Stimulation of in vitro angiogenesis by hydrogen peroxide and the relation with Ets-1 in endothelial cells. Life Sci..

[143] Qiu S.Q., Waaijer S.J., Zwager M.C., de Vries E.G., van der Vegt B., Schröder C.P. (2018). Tumor-associated macrophages in breast cancer: Innocent bystander or important player?. Cancer Treat. Rev..

[144] Pollard J.W. (2008). Macrophages define the invasive microenvironment in breast cancer. J. Leukoc. Biol..

[145] Choi J., Gyamfi J., Jang H., Koo J.S. (2018). The role of tumor-associated macrophage in breast cancer biology. Histol. Histopathol..

[146] Nandi B., Shapiro M., Samur M.K., Pai C., Frank N.Y., Yoon C., Prabhala R.H., Munshi N.C., Gold J.S. (2016). Stromal CCR6 drives tumor growth in a murine transplantable colon cancer through recruitment of tumor-promoting macrophages. OncoImmunology.

[147] Mantovani A., Marchesi F., Malesci A., Laghi L., Allavena P. (2017). Tumour-associated macrophages as treatment targets in oncology. Nat. Rev. Clin. Oncol..

[148] Lee C.H., Wu C.L., Shiau A.L. (2010). Toll-like Receptor 4 Signaling Promotes Tumor Growth. J. Immunother..

[149] Mehta A.K., Kadel S., Townsend M.G., Oliwa M., Guerriero J.L. (2021). Macrophage Biology and Mechanisms of Immune Suppression in Breast Cancer. Front. Immunol..

[150] Kalinski P. (2011). Regulation of Immune Responses by Prostaglandin E2. J. Immunol..

[151] Chen Y., Song Y., Du W., Gong L., Chang H., Zou Z. (2019). Tumor-associated macrophages: An accomplice in solid tumor progression. J. Biomed. Sci..

[152] Franklin R.A., Liao W., Sarkar A., Kim M.V., Bivona M.R., Liu K., Pamer E.G., Li M.O. (2014). The cellular and molecular origin of tumor-associated macrophages. Science.

[153] Martinez F.O., Gordon S., Locati M., Mantovani A. (2006). Transcriptional Profiling of the Human Monocyte-to-Macrophage Differentiation and Polarization: New Molecules and Patterns of Gene Expression. J. Immunol..

[154] Jung K.Y., Cho S.W., Kim Y.A., Kim D., Oh B.C., Park D.J., Park Y.J. (2015). Cancers with Higher Density of Tumor-Associated Macrophages Were Associated with Poor Survival Rates. J. Pathol. Transl. Med..

[155] Berger A. (2000). Science commentary: Th1 and Th2 responses: What are they?. BMJ.

[156] Sica A., Mantovani A. (2012). Macrophage plasticity and polarization: In vivo veritas. J. Clin. Investig..

[157] Ávila-Román J., García-Gil S., Rodríguez-Luna A., Motilva V., Talero E. (2021). Anti-Inflammatory and Anticancer Effects of Microalgal Carotenoids. Mar. Drugs.

[158] Kim K.N., Heo S.J., Yoon W.J., Kang S.M., Ahn G., Yi T.H., Jeon Y.J. (2010). Fucoxanthin inhibits the inflammatory response by suppressing the activation of NF-κB and MAPKs in lipopolysaccharide-induced RAW 264.7 macrophages. Eur. J. Pharmacol..

[159] Taciak B., Białasek M., Braniewska A., Sas Z., Sawicka P., Kiraga U., Rygiel T., Król M. (2018). Evaluation of phenotypic and functional stability of RAW 264.7 cell line through serial passages. PLoS ONE.

[160] Fuentes A.L., Millis L., Vapenik J., Sigola L. (2014). Lipopolysaccharide-mediated enhancement of zymosan phagocytosis by RAW 264.7 macrophages is independent of opsonins, laminarin, mannan, and complement receptor 3. J. Surg. Res..

[161] Satomi Y., Nishino H. (2013). Inhibition of the enzyme activity of cytochrome P450 1A1, 1A2 and 3A4 by fucoxanthin, a marine carotenoid. Oncol. Lett..

[162] Zanger U.M., Schwab M. (2013). Cytochrome P450 enzymes in drug metabolism: Regulation of gene expression, enzyme activities, and impact of genetic variation. Pharmacol. Ther..

[163] Desta Z., Kreutz Y., Nguyen A.T., Li L., Skaar T., Kamdem L.K., Henry N.L., Hayes D.F., Storniolo A.M., Stearns V. (2011). Plasma Letrozole Concentrations in Postmenopausal Women with Breast Cancer Are Associated with CYP2A6 Genetic Variants, Body Mass Index, and Age. Clin. Pharmacol. Ther..

[164] Gonzalez F.J., Gelboin H.V. (1994). Role of Human Cytochromes P450 in the Metabolic Activation of Chemical Carcinogens and Toxins. Drug Metab. Rev..

[165] Manikandan P., Nagini S. (2018). Cytochrome P450 Structure, Function and Clinical Significance: A Review. Curr. Drug Targets.

[166] Shimada T. (2006). Xenobiotic-Metabolizing Enzymes Involved in Activation and Detoxification of Carcinogenic Polycyclic Aromatic Hydrocarbons. Drug Metab. Pharmacokinet..

[167] Ding X., Kaminsky L.S. (2003). Human Extrahepatic Cytochromes P450: Function in Xenobiotic Metabolism and Tissue-Selective Chemical Toxicity in the Respiratory and Gastrointestinal Tracts. Annu. Rev. Pharmacol. Toxicol..

[168] Schneider E., Clark D.S. (2013). Cytochrome P450 (CYP) enzymes and the development of CYP biosensors. Biosens. Bioelectron..

[169] Murray G.I. (2000). The role of cytochrome P450 in tumour development and progression and its potential in therapy. J. Pathol..

[170] Luo B., Yan D., Yan H., Yuan J. (2021). Cytochrome P450: Implications for human breast cancer (Review). Oncol. Lett..

[171] McDaniel D.O., Thurber T., Lewis-Traylor A., Berry C., Barber W.H., Zhou X., Bigler S., Vance R. (2011). Differential Association of Cytochrome P450 3A4 Genotypes with Onsets of Breast Tumors in African American Versus Caucasian Patients. J. Investig. Med..

[172] Johnson N., Dudbridge F., Orr N., Gibson L., Jones M.E., Schoemaker M.J., Folkerd E.J., Haynes B.P., Hopper J.L., Southey M.C. (2014). Genetic variation at CYP3A is associated with age at menarche and breast cancer risk: A case-control study. Breast Cancer Res..

[173] Johnson N., Walker K., Gibson L.J., Orr N., Folkerd E., Haynes B., Palles C., Coupland B., Schoemaker M., Jones M. (2012). CYP3A Variation, Premenopausal Estrone Levels, and Breast Cancer Risk. JNCI J. Natl. Cancer Inst..

[174] Sangrajrang S., Sato Y., Sakamoto H., Ohnami S., Laird N.M., Khuhaprema T., Brennan P., Boffetta P., Yoshida T. (2009). Genetic polymorphisms of estrogen metabolizing enzyme and breast cancer risk in Thai women. Int. J. Cancer.

[175] Bai X., Xie J., Sun S., Zhang X., Jiang Y., Pang D. (2017). The associations of genetic polymorphisms in CYP1A2 and CYP3A4 with clinical outcomes of breast cancer patients in northern China. Oncotarget.

[176] Yim S.K., Kim K., Chun S., Oh T., Jung W., Jung K., Yun C.H. (2020). Screening of Human CYP1A2 and CYP3A4 Inhibitors from Seaweed in Silico and In Vitro. Mar. Drugs.

[177] Nebert D.W., Wikvall K., Miller W.L. (2013). Human cytochromes P450 in health and disease. Philos. Trans. R. Soc. B Biol. Sci..

[178] Tan B.S., Tiong K.H., Muruhadas A., Randhawa N., Choo H.L., Bradshaw T.D., Stevens M.F., Leong C.O. (2011). CYP2S1 and CYP2W1 Mediate 2-(3,4-Dimethoxyphenyl)-5-Fluorobenzothiazole (GW-610, NSC 721648) Sensitivity in Breast and Colorectal Cancer Cells. Mol. Cancer Ther..

[179] Cardenas-Rodriguez N., Lara-Padilla E., Bandala C., Lopez-Cruz J., Uscanga-Carmona C., Lucio-Monter P., Floriano-Sanchez E. (2012). CYP2W1, CYP4F11 and CYP8A1 Polymorphisms and Interaction of CYP2W1 Genotypes with Risk Factors in Mexican Women with Breast Cancer. Asian Pac. J. Cancer Prev..

[180] Moorthy B., Chu C., Carlin D.J. (2015). Polycyclic Aromatic Hydrocarbons: From Metabolism to Lung Cancer. Toxicol. Sci..

[181] Goth-Goldstein R. (2000). Interindividual variation in CYP1A1 expression in breast tissue and the role of genetic polymorphism. Carcinogenesis.

[182] Nilsson R., Antić R., Berni A., Dallner G., Dettbarn G., Gromadzinska J., Joksić G., Lundin C., Palitti F., Prochazka G. (2013). Exposure to polycyclic aromatic hydrocarbons in women from Poland, Serbia and Italy—Relation between PAH metabolite excretion, DNA damage, diet and genotype (the EU DIEPHY project). Biomarkers.

[183] Al-Dhfyan A., Alhoshani A., Korashy H.M. (2017). Aryl hydrocarbon receptor/cytochrome P450 1A1 pathway mediates breast cancer stem cells expansion through PTEN inhibition and β-Catenin and Akt activation. Mol. Cancer.

[184] Lin J.H. (2006). CYP Induction-Mediated Drug Interactions: In Vitro Assessment and Clinical Implications. Pharm. Res..

[185] Li W., Zhang H., Assaraf Y.G., Zhao K., Xu X., Xie J., Yang D.H., Chen Z.S. (2016). Overcoming ABC transporter-mediated multidrug resistance: Molecular mechanisms and novel therapeutic drug strategies. Drug Resist. Updates.

[186] Dean M. (2001). The Human ATP-Binding Cassette (ABC) Transporter Superfamily. Genome Res..

[187] Hollenstein K., Dawson R.J., Locher K.P. (2007). Structure and mechanism of ABC transporter proteins. Curr. Opin. Struct. Biol..

[188] Ween M., Armstrong M., Oehler M., Ricciardelli C. (2015). The role of ABC transporters in ovarian cancer progression and chemoresistance. Crit. Rev. Oncol. Hematol..

[189] Robey R.W., Pluchino K.M., Hall M.D., Fojo A.T., Bates S.E., Gottesman M.M. (2018). Revisiting the role of ABC transporters in multidrug-resistant cancer. Nat. Rev. Cancer.

[190] Ambudkar S.V., Kimchi-Sarfaty C., Sauna Z.E., Gottesman M.M. (2003). P-glycoprotein: From genomics to mechanism. Oncogene.

[191] Szakács G., Paterson J.K., Ludwig J.A., Booth-Genthe C., Gottesman M.M. (2006). Targeting multidrug resistance in cancer. Nat. Rev. Drug Discov..

[192] Waghray D., Zhang Q. (2017). Inhibit or Evade Multidrug Resistance P-Glycoprotein in Cancer Treatment. J. Med. Chem..

[193] He J., Fortunati E., Liu D.X., Li Y. (2021). Pleiotropic Roles of ABC Transporters in Breast Cancer. Int. J. Mol. Sci..

[194] Eid S.Y., El-Readi M.Z., Wink M. (2012). Carotenoids reverse multidrug resistance in cancer cells by interfering with ABC-transporters. Phytomedicine.

[195] Shin J., Song M.H., Oh J.W., Keum Y.S., Saini R.K. (2020). Pro-oxidant Actions of Carotenoids in Triggering Apoptosis of Cancer Cells: A Review of Emerging Evidence. Antioxidants.

[196] Black H.S., Boehm F., Edge R., Truscott T.G. (2020). The Benefits and Risks of Certain Dietary Carotenoids that Exhibit both Anti- and Pro-Oxidative Mechanisms—A Comprehensive Review. Antioxidants.

[197] Vijay K., Sowmya PR R., Arathi B.P., Shilpa S., Shwetha H.J., Raju M., Baskaran V., Lakshminarayana R. (2018). Low-dose doxorubicin with carotenoids selectively alters redox status and upregulates oxidative stress-mediated apoptosis in breast cancer cells. Food Chem. Toxicol..

[198] Muftah A.A., Aleskandarany M.A., Al-kaabi M.M., Sonbul S.N., Diez-Rodriguez M., Nolan C.C., Caldas C., Ellis I.O., Rakha E.A., Green A.R. (2017). Ki67 expression in invasive breast cancer: The use of tissue microarrays compared with whole tissue sections. Breast Cancer Res. Treat..

[199] Nahed A.S., Shaimaa M.Y. (2016). Ki-67 as a prognostic marker according to breast cancer molecular subtype. Cancer Biol. Med..

[200] Fischer U., Schulze-Osthoff K. (2005). Apoptosis-based therapies and drug targets. Cell Death Differ..

[201] Chen D.L., Engle J.T., Griffin E.A., Miller J.P., Chu W., Zhou D., Mach R.H. (2014). Imaging Caspase-3 Activation as a Marker of Apoptosis-Targeted Treatment Response in Cancer. Mol. Imaging Biol..

[202] Devarajan E., Sahin A.A., Chen J.S., Krishnamurthy R.R., Aggarwal N., Brun A.M., Sapino A., Zhang F., Sharma D., Yang X.H. (2002). Down-regulation of caspase 3 in breast cancer: A possible mechanism for chemoresistance. Oncogene.

[203] Kalaitzidis D., Gilmore T.D. (2005). Transcription factor cross-talk: The estrogen receptor and NF-κB. Trends Endocrinol. Metab..

[204] Frasor J., Weaver A., Pradhan M., Dai Y., Miller L.D., Lin C.Y., Stanculescu A. (2009). Positive Cross-Talk between Estrogen Receptor and NF-κB in Breast Cancer. Cancer Res..

[205] Zubair A., Frieri M. (2012). Role of Nuclear Factor-κB in Breast and Colorectal Cancer. Current Allergy Asthma Rep..

[206] Cogswell P.C., Guttridge D.C., Funkhouser W.K., Baldwin A.S. (2000). Selective activation of NF-κB subunits in human breast cancer: Potential roles for NF-κB2/p52 and for Bcl-3. Oncogene.

[207] Guo J., Verma U.N., Gaynor R.B., Frenkel E.P., Becerra C.R. (2004). Enhanced Chemosensitivity to Irinotecan by RNA Interference-Mediated Down-Regulation of the Nuclear Factor-κB p65 Subunit. Clin. Cancer Res..

[208] Pan X., Arumugam T., Yamamoto T., Levin P.A., Ramachandran V., Ji B., Lopez-Berestein G., Vivas-Mejia P.E., Sood A.K., McConkey D.J. (2008). Nuclear Factor- B p65/relA Silencing Induces Apoptosis and Increases Gemcitabine Effectiveness in a Subset of Pancreatic Cancer Cells. Clin. Cancer Res..

[209] Wang B., Wei H., Prabhu L., Zhao W., Martin M., Hartley A.V., Lu T. (2015). Role of Novel Serine 316 Phosphorylation of the p65 Subunit of NF-κB in Differential Gene Regulation. J. Biol. Chem..

[210] Hongisto V., Jernström S., Fey V., Mpindi J.P., Kleivi Sahlberg K., Kallioniemi O., Perälä M. (2013). High-Throughput 3D Screening Reveals Differences in Drug Sensitivities between Culture Models of JIMT1 Breast Cancer Cells. PLoS ONE.

[211] Lovitt C.J., Shelper T.B., Avery V.M. (2018). Doxorubicin resistance in breast cancer cells is mediated by extracellular matrix proteins. BMC Cancer.

[212] Lobo V., Patil A., Phatak A., Chandra N. (2010). Free radicals, antioxidants and functional foods: Impact on human health. Pharmacogn. Rev..

[213] Commoner B., Townsend J., Pake G.E. (1954). Free Radicals in Biological Materials. Nature.

[214] Simic M.G., Bergtold D.S., Karam L.R. (1989). Generation of oxy radicals in biosystems. Mutat. Res. Fundam. Mol. Mech. Mutagenesis.

[215] Darley-Usmar V., Halliwell B. (1996). Blood radicals: Reactive nitrogen species, reactive oxygen species, transition metal ions, and the vascular system. Pharm. Res..

[216] Heffernan N., Smyth T.J., FitzGerald R.J., Soler-Vila A., Brunton N. (2014). Antioxidant activity and phenolic content of pressurised liquid and solid-liquid extracts from four Irish origin macroalgae. Int. J. Food Sci. Technol..

[217] Nomura T., Kikuchi M., Kubodera A., Kawakami Y. (1997). Proton-donative antioxidant activity of fucoxanthin with 1,1-Diphenyl-2-Picrylhydrazyl (DPPH). IUBMB Life.

[218] Yan X., Chuda Y., Suzuki M., Nagata T. (1999). Fucoxanthin as the Major Antioxidant inHijikia fusiformis, a Common Edible Seaweed. Biosci. Biotechnol. Biochem..

[219] Zaragozá M.C., López D., Sáiz M.P., Poquet M., Pérez J., Puig-Parellada P., Màrmol F., Simonetti P., Gardana C., Lerat Y. (2008). Toxicity and Antioxidant Activity in Vitro and in Vivo of Two *Fucus vesiculosus* Extracts. J. Agric. Food Chem..

[220] Miller N.J., Sampson J., Candeias L.P., Bramley P.M., Rice-Evans C.A. (1996). Antioxidant activities of carotenes and xanthophylls. FEBS Lett..

[221] Mortensen A., Skibsted L.H., Sampson J., Rice-Evans C., Everett S.A. (1997). Comparative mechanisms and rates of free radical scavenging by carotenoid antioxidants. FEBS Lett..

[222] Aggarwal V., Tuli H., Varol A., Thakral F., Yerer M., Sak K., Varol M., Jain A., Khan M., Sethi G. (2019). Role of Reactive Oxygen Species in Cancer Progression: Molecular Mechanisms and Recent Advancements. Biomolecules.

[223] World Health Organization (WHO) Obesity and Overweight. https://www.who.int/news-room/fact-sheets/detail/obesity-and-overweight.

[224] Engin A. (2017). Obesity-associated Breast Cancer: Analysis of risk factors. Adv. Exp. Med. Biol..

[225] Barone I., Giordano C., Bonofiglio D., Andò S., Catalano S. (2020). The weight of obesity in breast cancer progression and metastasis: Clinical and molecular perspectives. Semin. Cancer Biol..

[226] Boyd N.F., McGuire V. (1990). Evidence of association between plasma high-density lipoprotein cholesterol and risk factors for breast cancer. J. Natl. Cancer Inst..

[227] McDonnell D.P., Chang C.-Y., Nelson E.R. (2014). The estrogen receptor as a mediator of the pathological actions of cholesterol in breast cancer. Climacteric J. Int. Menopause Soc..

[228] Garcia-Estevez L., Moreno-Bueno G. (2019). Updating the role of obesity and cholesterol in breast cancer. Breast Cancer Res. BCR.

[229] Kerr J., Anderson C., Lippman S.M. (2017). Physical activity, sedentary behaviour, diet, and cancer: An update and emerging new evidence. Lancet Oncol..

[230] Yang X.R., Chang-Claude J., Goode E.L., Couch F.J., Nevanlinna H., Milne R.L., Gaudet M., Schmidt M.K., Broeks A., Cox A. (2011). Associations of breast cancer risk factors with tumor subtypes: A pooled analysis from the Breast Cancer Association Consortium studies. J. Natl. Cancer Inst..

[231] Munsell M.F., Sprague B.L., Berry D.A., Chisholm G., Trentham-Dietz A. (2014). Body mass index and breast cancer risk according to postmenopausal estrogen-progestin use and hormone receptor status. Epidemiol. Rev..

[232] Neuhouser M.L., Aragaki A.K., Prentice R.L., Manson J.E., Chlebowski R., Carty C.L., Ochs-Balcom H.M., Thomson C.A., Caan B.J., Tinker L.F. (2015). Overweight, Obesity, and Postmenopausal Invasive Breast Cancer Risk: A Secondary Analysis of the Women’s Health Initiative Randomized Clinical Trials. JAMA Oncol..

[233] Nagrani R., Mhatre S., Rajaraman P., Soerjomataram I., Boffetta P., Gupta S., Parmar V., Badwe R., Dikshit R. (2016). Central obesity increases risk of breast cancer irrespective of menopausal and hormonal receptor status in women of South Asian Ethnicity. Eur. J. Cancer.

[234] Ritte R., Lukanova A., Berrino F., Dossus L., Tjønneland A., Olsen A., Overvad T.F., Overvad K., Clavel-Chapelon F., Fournier A. (2012). Adiposity, hormone replacement therapy use and breast cancer risk by age and hormone receptor status: A large prospective cohort study. Breast Cancer Res..

[235] Chan D., Vieira A.R., Aune D., Bandera E.V., Greenwood D.C., McTiernan A., Navarro Rosenblatt D., Thune I., Vieira R., Norat T. (2014). Body mass index and survival in women with breast cancer-systematic literature review and meta-analysis of 82 follow-up studies. Ann. Oncol. Off. J. Eur. Soc. Med. Oncol..

[236] Fortner R.T., Katzke V., Kühn T., Kaaks R. (2016). Obesity and Breast Cancer. Recent results in cancer research. Obesity Cancer.

[237] Miyashita K., Nishikawa S., Beppu F., Tsukui T., Abe M., Hosokawa M. (2011). The allenic carotenoid fucoxanthin, a novel marine nutraceutical from brown seaweeds. J. Sci. Food Agric..

[238] Wu J., Boström P., Sparks L., Ye L., Choi J., Giang A.H., Khandekar M., Virtanen K., Nuutila P., Schaart G. (2012). Beige Adipocytes Are a Distinct Type of Thermogenic Fat Cell in Mouse and Human. Cell.

[239] Gammone M., D’Orazio N. (2015). Anti-Obesity Activity of the Marine Carotenoid Fucoxanthin. Mar. Drugs.

[240] Pangestuti R., Siahaan E.A., Qin Y. (2018). Seaweed-derived carotenoids. Bioactive Seaweeds for Food Applications.

[241] Ikeda K., Yamada T. (2020). UCP1 Dependent and Independent Thermogenesis in Brown and Beige Adipocytes. Front. Endocrinol..

[242] Madsen L., Pedersen L.M., Lillefosse H.H., Fjære E., Bronstad I., Hao Q., Petersen R.K., Hallenborg P., Ma T., de Matteis R. (2010). UCP1 Induction during Recruitment of Brown Adipocytes in White Adipose Tissue Is Dependent on Cyclooxygenase Activity. PLoS ONE.

[243] Vegiopoulos A., Müller-Decker K., Strzoda D., Schmitt I., Chichelnitskiy E., Ostertag A., Diaz M.B., Rozman J., Hrabe De Angelis M., Nüsing R.M. (2010). Cyclooxygenase-2 Controls Energy Homeostasis in Mice by de Novo Recruitment of Brown Adipocytes. Science.

[244] Hosokawa M., Miyashita T., Nishikawa S., Emi S., Tsukui T., Beppu F., Okada T., Miyashita K. (2010). Fucoxanthin regulates adipocytokine mRNA expression in white adipose tissue of diabetic/obese KK-Ay mice. Arch. Biochem. Biophys..

[245] Kang S.I., Shin H.S., Kim H.M., Yoon S.A., Kang S.W., Kim J.H., Ko H.C., Kim S.J. (2012). *Petalonia binghamiae* Extract and Its Constituent Fucoxanthin Ameliorate High-Fat Diet-Induced Obesity by Activating AMP-Activated Protein Kinase. J. Agric. Food Chem..

[246] Woo M.N., Jeon S.M., Kim H.J., Lee M.K., Shin S.K., Shin Y.C., Park Y.B., Choi M.S. (2010). Fucoxanthin supplementation improves plasma and hepatic lipid metabolism and blood glucose concentration in high-fat fed C57BL/6N mice. Chem. Biol. Interact..

[247] Ha A.W., Kim W.K. (2013). The effect of fucoxanthin rich power on the lipid metabolism in rats with a high fat diet. Nutr. Res. Pract..

[248] Abidov M., Ramazanov Z., Seifulla R., Grachev S. (2010). The effects of Xanthigen in the weight management of obese premenopausal women with non-alcoholic fatty liver disease and normal liver fat. Diabetes Obes. Metab..

[249] Jeon S.M., Kim H.J., Woo M.N., Lee M.K., Shin Y.C., Park Y.B., Choi M.S. (2010). Fucoxanthin-rich seaweed extract suppresses body weight gain and improves lipid metabolism in high-fat-fed C57BL/6J mice. Biotechnol. J..

[250] Cheng J., Fujita A., Ohsaki Y., Suzuki M., Shinohara Y., Fujimoto T. (2009). Quantitative electron microscopy shows uniform incorporation of triglycerides into existing lipid droplets. Histochem. Cell Biol..

[251] Zhang S., Hunter D.J., Forman M.R., Rosner B.A., Speizer F.E., Colditz G.A., Manson J.E., Hankinson S.E., Willett W.C. (1999). Dietary Carotenoids and Vitamins A, C, and E and Risk of Breast Cancer. JNCI J. Natl. Cancer Inst..

[252] McTiernan A. (2021). Dietary prevention of breast cancer in high-risk women: Role of carotenoids. Am. J. Clin. Nutr..

[253] Dandona P., Aljada A., Chaudhuri A., Mohanty P., Garg R. (2005). Metabolic Syndrome. Circulation.

[254] Rupnick M.A., Panigrahy D., Zhang C.Y., Dallabrida S.M., Lowell B.B., Langer R., Folkman M.J. (2002). Adipose tissue mass can be regulated through the vasculature. Proc. Natl. Acad. Sci. USA.

